# Recent Advances in In-Memory Computing: Exploring Memristor and Memtransistor Arrays with 2D Materials

**DOI:** 10.1007/s40820-024-01335-2

**Published:** 2024-02-19

**Authors:** Hangbo Zhou, Sifan Li, Kah-Wee Ang, Yong-Wei Zhang

**Affiliations:** 1 Institute of High Performance Computing (IHPC), Agency for Science, Technology and Research (A*STAR), 1 Fusionopolis Way, #16-16 Connexis, Singapore, 138632 Republic of Singapore; 2 Department of Electrical and Computer Engineering, National University of Singapore, 4 Engineering Drive 3, Singapore, 117583 Republic of Singapore; 3 Institute of Materials Research and Engineering, Agency for Science, Technology and Research (A*STAR), 2 Fusionopolis Way, Singapore, 138634 Republic of Singapore

**Keywords:** 2D materials, Memristors, Memtransistors, Crossbar array, In-memory computing

## Abstract

The conventional computing architecture faces substantial challenges, including high latency and energy consumption between memory and processing units. In response, in-memory computing has emerged as a promising alternative architecture, enabling computing operations within memory arrays to overcome these limitations. Memristive devices have gained significant attention as key components for in-memory computing due to their high-density arrays, rapid response times, and ability to emulate biological synapses. Among these devices, two-dimensional (2D) material-based memristor and memtransistor arrays have emerged as particularly promising candidates for next-generation in-memory computing, thanks to their exceptional performance driven by the unique properties of 2D materials, such as layered structures, mechanical flexibility, and the capability to form heterojunctions. This review delves into the state-of-the-art research on 2D material-based memristive arrays, encompassing critical aspects such as material selection, device performance metrics, array structures, and potential applications. Furthermore, it provides a comprehensive overview of the current challenges and limitations associated with these arrays, along with potential solutions. The primary objective of this review is to serve as a significant milestone in realizing next-generation in-memory computing utilizing 2D materials and bridge the gap from single-device characterization to array-level and system-level implementations of neuromorphic computing, leveraging the potential of 2D material-based memristive devices.

## Highlights

- State-of-the-art research on two-dimensional material-based memristive arrays is comprehensively reviewed.
- Critical steps in achieving in-memory computing are identified and highlighted, covering material selection, device performance analysis, and array structure design.
- Challenges in progressing from single-device characterization to array-level and system-level implementations are discussed, along with proposed solutions.

## Graphical abstract

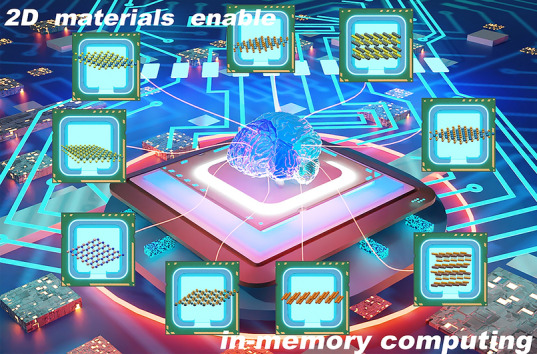

## Introduction

In the post-Moore's law era, the traditional von Neumann architecture has reached its limits in terms of computational capability per energy consumed, leading to a considerable slowdown in improvement. However, the demand for computational power, especially in the field of artificial intelligence, has soared while energy consumption has become a pressing concern. Consequently, there has been a growing momentum towards energy-efficient computing, also known as "green computing", and in-memory computing has emerged as a promising solution to address these critical challenges. In-memory computing eliminates the need to transfer data between memory and processing units, thereby significantly reducing energy consumption. Neuromorphic computing, inspired by the human brain's functionality, stands out as an exemplar of in-memory computing that achieves remarkable levels of energy efficiency [1–6].

In-memory computing can be built upon transistors and memristive devices. Transistor-based in-memory computing chips, such as static random-access memory (SRAM), dynamic random-access memory (DRAM), and floating gate memory, are well-suited for logic computing that requires precise storage and processing of data. However, these devices have limitations in terms of their power consumption and response time [7–10]. A significant challenge associated with SRAM is its large footprint, ranging from 123 to 140 F^2^, leading to challenges in chip area downscaling when constructing SRAM-based crossbar arrays. Furthermore, scaling the area becomes even more challenging when analog in-memory computing is required. This is because SRAM can only store binary data in a single cell, necessitating the stacking of multiple cells to represent multi-bit data. Additionally, owing to the low transistor barrier height (0.5 eV), the charges within SRAM are volatile and constantly require refreshment from an external power source, resulting in additional standby power consumption. While DRAM boasts a smaller footprint (6 F^2^), it remains a volatile memory that necessitates periodic refreshing (every 60 ms) to prevent loss of charges due to leakage current and cell readings. This cell refreshing process also contributes to increased power consumption within the DRAM crossbar array. Meanwhile, although being a non-volatile memory, the floating gate transistor suffers from a slow response speed (> 10 µs) and high functional voltage (> 10 V).

In contrast, memristive devices, which include memristors and memtransistors, are particularly suitable for neuromorphic computing [11]. A memristor is a passive electrical component known for its resistive switching behavior. Its resistance changes based on the history of electric current passing through it, resulting in a memory effect. A memtransistor, on the other hand, is a hybrid device that combines the characteristics of a memristor and a transistor. Memtransistors feature a gate terminal, enabling the modulation of their resistive switching behavior. They offer several advantages over transistor-based technologies, such as low power consumption, support for analog computing, and the ability to perform massive parallelism in simulating neural networks. Additionally, memristive devices enable new computing paradigms that are difficult to emulate using traditional architectures, such as the spiking neural network [12]. Specifically, memristive devices excel at matrix multiplication and accumulation (MAC), one of the most computationally intensive operations in conventional digital computing and extensively required in artificial neural network computing [13–15]. Therefore, the exploration and advancement of memristive devices hold great promise for next-generation energy-efficient computing.

The development of memristive devices has been rapid in recent years [16–19]. The first memristor was experimentally demonstrated in 2008 using metal oxides [20, 21], which switch between high-resistance and low-resistance states due to oxygen migration. Since then, researchers have made rapid progress in developing memristors based on differential metals such as titanium oxide, tantalum oxide, and hafnium oxide [22, 23], as well as phase-change materials such as germanium-antimony-tellurium [24, 25]. Despite these advances, there are several challenges in meeting industrial requirements. For example, memristors based on metal oxide materials exhibit high device variation, which makes it difficult for precise control over resistance switching [26–29]. In addition, phase-change devices require high energy and long program duration for crystal structure transition, which limits their compatibility with advanced complementary metal–oxide–semiconductor (CMOS) technology [30].

Recently, memristors made up of two-dimensional (2D) materials have emerged as a promising area of research [31–36]. 2D materials offer several advantages as functional materials for memristors, such as low switching voltage, reduced power consumption due to their ultra-thin body [34, 37–42], and absence of dangling bonds that can cause scalability issues with ultrathin oxides [43]. The ultrathin body of the 2D semiconductor channel allows for precise control of the gate voltage and potential immunity to the short-channel effect, making it possible to create multiterminal memtransistors [31, 44–46]. Additionally, the abundance and stackable nature of 2D materials enables the creation of van der Waals heterostructures by combining different 2D materials in a designed order, overcoming limitations related to lattice matching or processing [36, 47–51]. Furthermore, the high surface-to-volume ratio of 2D materials allows for excellent sensing capabilities [52, 53], and they exhibit properties such as flexibility, shorter response time, and broader temperature ranges for device operation [34, 48, 54–56].

In this review, we will focus exclusively on the advances made in the past three years (since 2019), which represent the current frontier of research in this field. Within this timeframe, we have witnessed not just the continuous development of single memristor and memtransistor devices, but even more importantly, the successful integration of devices and the realization of crossbar arrays in experimental settings. This crucial milestone marks a significant step forward in the direction of achieving artificial neural networks and functional devices with potential commercial applications in the future. It is noteworthy that such advancements were not evident three years ago, when the primary research emphasis was still on individual devices, and array performance was largely reliant on simulations. Consequently, our review centers on these crossbars realized memristive devices and meticulously assesses their performance within the context of integrated arrays. For a wider perspective on memristive device performance based on 2D materials, readers may refer to the extensive reviews in the literature [31, 57–63]. Those keen on exploring the crossbar array performance based on numerical simulation may also refer to the reviews [9, 64, 65].

**Fig. 1:**
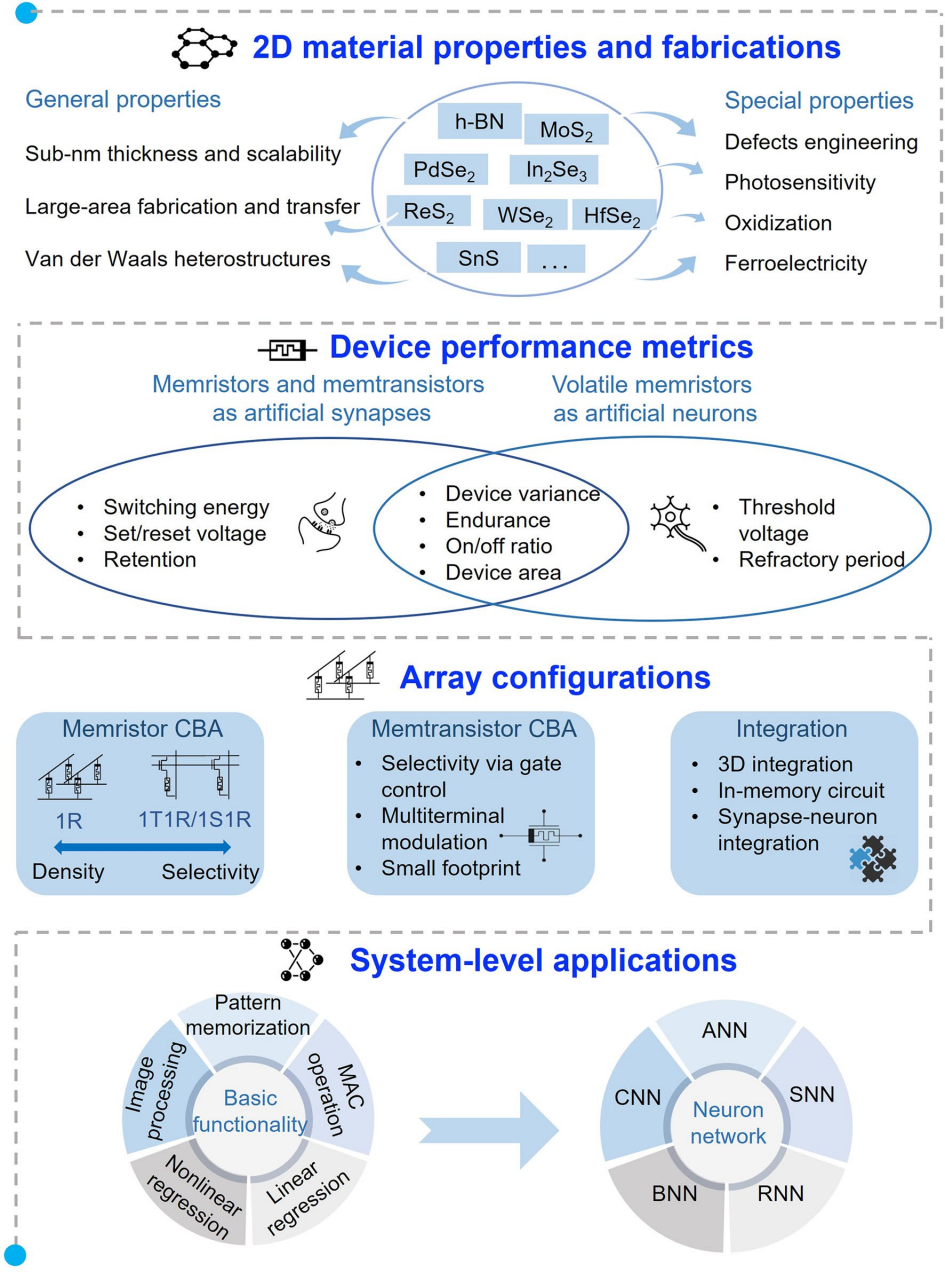
A schematic of critical steps in 2D material-based in-memory computing applications. First, unique 2D material properties and the fundamental memristive device fabrication and switching mechanisms. Second, different device performance requirements for various applications, including artificial synapses and neurons. Third, different array configurations for integration design, including memristor and memtransistor crossbar array and 3D integration. Last, system-level evaluation of in-memory computing hardware, consisting of the basic computation functionalities and the overall neural network performance

This review will be organized as shown in Fig. 1. We will begin with a summary of the 2D material platforms that have been developed for memristive device array fabrication. Subsequently, we will discuss the application-dependent device performance metrics of 2D material-based memristive devices in arrays. We will also explore various array structures that have been demonstrated to achieve in-memory computing functionalities. Furthermore, we will examine the potential computing applications based on these arrays, including neural networks and information processing. Finally, we will provide a brief overview of the current challenges and potential solutions for the development of 2D material-based memristive arrays and their system-level implementation of in-memory computing.

## 2D Material Platforms for Memristive Device Array Fabrication

2D materials exhibit several shared characteristics that render them highly suitable for the fabrication of memristive devices, particularly for device arrays.

Firstly, the monolayer structure of functional 2D materials reduces device thickness to the sub-nanometer scale, allowing for exceptional scalability and controllability in the creation of high-density three-dimensional (3D) crossbar array [15, 66, 67]. For example, Wu et al*.* reported a monolayer h-BN-based non-volatile memory with a record thickness of 0.33 nm [42]. Additionally, in memtransistor devices based on 2D materials like molybdenum disulfide, the ultra-thin atomic layer permits effective gate control in multiterminal devices, leading to enhanced device selection in crossbar arrays [68].

Secondly, the capability of large-area wafer-based fabrication and uniform thickness transfer has been successfully demonstrated for many 2D materials, including the well-studied ones like hexagonal boron nitride [55, 69], molybdenum disulfide [70]. Recently, Li et al*.* have developed a wafer-scale growth technique for HfSe_2_ using molecular beam epitaxy (MBE) in conjunction with a metal-assisted van der Waals transfer process [71]. These advanced fabrications and transfer techniques empower the fabrication of large-scale memristor crossbar arrays utilizing 2D materials as functional components.

Lastly, the layered structure of 2D materials, with weak interactions between layers and an absence of dangling bonds, opens up abundant possibilities for forming functional heterostructures and achieving effective electrode contract in the design of memristive devices [59].

In addition to these shared characteristics, various 2D materials possess unique properties that make them well-suited for designing memristive devices. These material-specific properties should be discussed together with the switching mechanisms of the memristors and memtransistors, as different switching mechanism will impose distinct requirements on the material properties. The switching mechanism of memristive devices based on 2D materials includes conductive filament formation [34, 51, 71–76], vacancy migration [44, 48, 77–80], photon responses [53, 81, 82], phase change [32, 83] and ferroelectricity [84, 85]. In the following sections, we will discuss the distinctive properties of various 2D materials within the context of the different switching mechanisms and analyze their specific advantages.

### Conductive Filament Formation: h-BN, MoS_2_, PdSe_2_ HfSe_2_ and BP

**Fig. 2:**
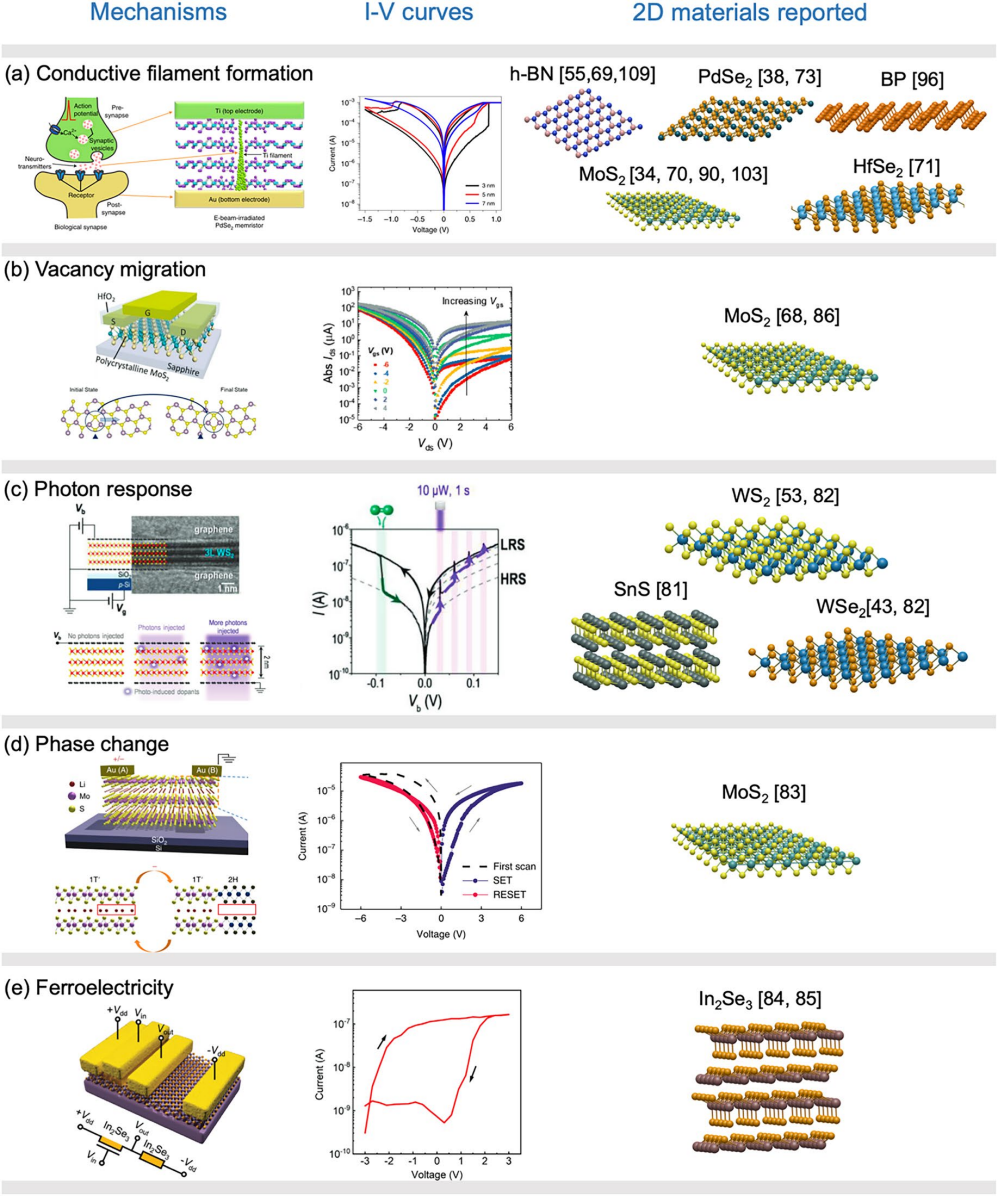
2D material platforms for memristors and memtransistors with different switching mechanisms: **a** conductive filament formation, **b** vacancy migration, **c** photon response, **d** phase change and **e** ferroelectricity. The first column shows the schematic representation of the respective resistive switching mechanisms, the second column displays the measured *I–V* curves and the final column presents the atomic structures of 2D materials that have been used for crossbar array fabrication. **a** Reproduced with permission [73], copyright © 2021 Springer Nature Limited. **b** Reproduced with permission [77], copyright © 2019 WILEY‐VCH Verlag GmbH & Co. KGaA, Weinheim. **c** Reproduced with permission [82], copyright © 2022 Wiley‐VCH GmbH. **d** Reproduced with permission [83], copyright © 2018 Springer Nature Limited. **e** Reproduced with permission [85], copyright © 2022 Wiley‐VCH GmbH

Conductive filament formation is a common switching mechanism in vertical memristors, where a 2D insulator or semiconductor is sandwiched between two metal electrodes, as shown in Fig. 2a. Initially, these devices exhibit a high-resistance state (HRS) due to the insulating layers. However, the application of an electric field can lead to the creation of a conducting filament between the two electrodes by allowing metal atoms to penetrate, causing the device to switch from HRS to a low-resistance state (LRS). This process is analogous to the release of neuro-transmitters in biological synapse. Reversing the electric field results in the rupture of the filament, returning the device from LRS to HRS. Figure 2a shows these two switching processes when palladium diselenide is placed between Ti and Au electrodes, with various palladium diselenide thicknesses. In some cases, the filament's rupture occurs spontaneously without the need for a reverse electric field, making the memristor volatile in nature.

*Hexagonal boron nitride (h-BN)* is a widely used as an insulating material for metal–insulator–metal (MIM) memristors due to its various advantageous properties. Firstly, h-BN possesses high insulation with a substantial bandgap of 5.9 eV, resulting in an initial high resistance state (HRS) for achieving large resistive switching (RS) ratios, which is a key consideration for large-scale crossbar array fabrication as it helps compensate for the sensing margin reduction caused by the array's leakage current [45, 86, 87]. Secondly, it exhibits high mechanical, chemical, and thermal stability across various thicknesses, ranging from multilayer to monolayer sheets. The high thermal stability of h-BN, stemming from strong boron nitride bond and low thermodynamic energy, ensures smooth and predictable relaxation processes when driven forces are removed. This enhances the retention characteristics of h-BN-based memristors [69]. In addition, its chemical inertness to oxidation and excellent adhesive properties to metals contributes to the structural stability of devices [88]. Last but importantly, Shen et al. have recently shown that the cross-plane conductance of h-BN-based memristors is dominated by the most conductive locations where a conductive path can be quickly established. At these specific defect sites, facilitated by the intrinsic defect region in the h-BN film, resulting in the low formation energy of filaments, results in a low set voltage and power consumption, leading to proposed switching energies at the zeptojoule level for h-BN memristors, significantly lower than those of other materials [55, 56, 69, 86]. Furthermore, the local conductivity overwrites the effects of other defects within the h-BN layer, so that they showcase low device-to-device variance and high yield. Shen et al*.* have shown that h-BN memristors can achieve a yield of around 50% even at a scale of 320 nm × 420 nm, overcoming most of the artifacts during the fabrication process, such as the thicker islands, impurity particles and wrinkles. Such scaling property is promising for high-density crossbar arrays and integration with CMOS transistors [89].

*Molybdenum disulfide (MoS*_*2*_*)* also received significant attention for filament-formation vertical memristors. These devices typically utilize MoS_2_ in the semiconducting 2H phase sandwiched between two electrodes. The advantage of MoS_2_ is attributed to its grain boundaries in polycrystalline MoS_2_ for a guided filament formation and hence provides better controllability. Feng et al*.* demonstrated a fully printed MoS_2_ memristor crossbar array, showing the existence of inter-layer grain boundaries in the printed MoS_2_ film [34]. Density functional theory (DFT) calculation and conductive-atomic force microscope (CAFM) confirmed the facilitating effect of grain boundaries on the migration of Ag atoms during filament formation and rupture. Additionally, Tang et al*.* revealed that the sulfur vacancies percolation along the flake edges of MoS_2_ is able to further modulate the resistance switching behavior [90]. The temperature-dependent measurements verified the change in barrier height during the transition between the LRS and HRS. Therefore, the switching behavior of vertical MoS_2_ memristors can be tailored by engineering the density of grain boundaries and the sulfur vacancy diffusion barrier at the edge.

*Palladium diselenide (PdSe*_*2*_*)* is another semiconductor material that offers guided conductive filament formation at grain boundaries. Selenide vacancies in PdSe_2_ play a crucial role as they exhibit a low diffusion barrier, facilitating local phase transitions of PdSe_2_ that resulted in the formation of heterophase grain boundaries. Notably, Li et al*.* recently fabricated a crossbar array comprising PdSe_2_-based memristors, wherein these heterophase grain boundaries were found to guide the formation of conductive filaments, resulting in resistive switching behavior with improved variability [73]. Furthermore, the utilization of PdSeO_x_/PdSe_2_ heterostructures in memristor crossbar arrays has also been demonstrated through ultraviolet-ozone treatment [38]. These devices exhibit ultra-thin switching media and uniform switching voltages, owing to the confinement of conductive filament formation within the heterostructure.

*Hafnium diselenide (HfSe*_*2*_*)* has also been utilized for filament-formation-based memristors [91, 92] and wafer-scale crossbar arrays [71]. The formation of metal-HfSe_2_ alloys at the electrodes contact exhibits variable resistance states, highlighting the potential for memristive devices [93, 94]. On the other hand, HfSe_2_ can be oxidized to form a high-resistance HfSe_x_O_y_ film, making it suitable for low-power devices. Recent demonstrations have shown memristors based on HfSe_x_O_y_ with a low switching energy of 114 fJ [95]. The as-grown polycrystalline HfSe_2_ exhibits no preferred in-plane orientation, suggesting the presence of intrinsic defects that could serve as pathways for conductive filament formation during resistive switching.

*Rhenium disulfide (ReS*_*2*_*)* has shown significant potential as a functional material for synthesizing crossbar arrays through the formation of metallic ReO_x_ through oxidation. However, the low formation energy is attributed to its sulfur vacancies [76]. An active approach for inducing sulfur vacancies into ReS_2_ involves Mo beam irradiation through MBE [76]. Both DFT calculations and experimental results have shown that the presence of sulfur vacancies in Mo-irradiated ReS_2_ leads to a lower energy barrier for the formation of metallic ReO_x_ filaments. The use of electron beam and Mo beam irradiation techniques offers effective methods for creating sulfur vacancies, opening up possibilities for the development of ReS_2_-based memristor arrays.

*Black phosphorene (BP)* is an appealing semiconductor with an optimal bandgap, high electron mobility, and substantial anisotropy. However, its susceptibility to oxidation in ambient air poses a challenge to its stability against oxidation. On the other hand, PO_x_ has high electric resistance and therefore could be used as insulating materials for filament-formation-based memristors. Wang et al*.* successfully fabricated a BP-based crossbar array for artificial synapses by incorporating an ultrathin interface layer of phosphorous oxides (PO_x_) on the BP surface [96]. The switching mechanism in this system is triggered by the migration of oxygen vacancies, leading to the formation and rupture of conductive filaments.

### Vacancy Migration: MoS_2_ and ReS_2_

Several 2D semiconductors exhibit a low diffusion barrier of intrinsic vacancies, often facilitated by the grain boundaries, as illustrated in Fig. 2b. At the metal–semiconductor contact region of these memristive devices, a Schottky barrier may form. The resistance is primarily governed by the electrons overcoming the Schottky barrier height (SBH), which, in turn, depends on the vacancy concentrations in the contact region. Consequently, the SBH can be modulated through the migration of vacancies [97]. For such switching mechanism, the resistance switches gradually in contrast to the abrupt change in filament-formation-based devices. An additional advantage of these devices is their capability to introduce other control terminals, such as gates, to form a multiterminal memtransistors [44, 68, 77, 79, 80, 86]. Figure 2b shows a schematic of a three-terminal memtransistors based on MoS_2_. We may observe that its *I–V* curves can be modulated through the gate terminal. The capability of modifying the resistance state through gate terminal demonstrates self-selective behavior and reduces sneak path currents [86].

*Molybdenum disulfide (MoS*_*2*_*)* offers an excellent candidate for such SBH-based memtransistors due to the low diffusion barrier of sulfur vacancies and effective control of SBH. DFT calculations have shown that sulfur vacancy is able to migrate in the perpendicular direction of grain boundaries with low diffusion barrier, resulting in a low set/reset voltage [77]. The presence of sulfur vacancies at the metal–MoS_2_ interfaces effectively influence the SBH through the hybridization of electronic states. First-principles calculations have shown that sulfur vacancies in MoS_2_ can increase the SBH when in contact with Mg, Al, and In, while decrease when in contact with Cu, Ag, Pd [98], Co and Ni [99]. Furthermore, sulfur vacancies create defect states near the interfaces that can be filled by electrons from the metal electrodes, resulting in Fermi-level pinning. Doping also introduces states that trap charges near the contacts, leading to a doping-induced reduction of the SBH [44, 68, 100].

*Rhenium disulfide (ReS*_*2*_*)* also shows similar SBH modulation attributed to its sulfur vacancies. Notably, the creation of sulfur vacancies can be actively achieved through electron beam irradiation, which enables the drifting of these vacancies under the influence of an electric field. This process further modulates the Schottky barrier height (SBH) at the contact of lateral ReS_2_-based memristors [78]. In contrast to MoS_2_ where the sulfur vacancies are positively charged [80], the sulfur vacancies in ReS_2_ are negatively charged, so that the vacancies migrate in the opposite direction under a given electric field.

### Photon Response: WSe_2_, WS_2_, and SnS

Semiconductors that have bandgap falling within the spectrum of visible light can be effectively employed for photodetector in sensors. Such semiconductors include WSe_2_, MoS_2_, WS_2_, and SnS. The properties of photosensitivity have been combined with the memristive circuits to realize unified sensor-processor devices. Figure 2c shows a scanning image and schematic representation of a photon response memristors. When exposed to the UV light illumination, tungsten disulfide (indicated by the pink lines in *I–V* curve) undergoes changes, resulting in the creation of defects such as sulfur vacancies, and hence, the device switches from HRS to LRS. Conversely, when exposed to oxygen environment, the device switches from LRS to HRS (indicated by the green lines in *I–V* curve) due to the incorporation of oxygen.

*Tungsten diselenide (WSe*_*2*_*) and tungsten disulfide (WS*_*2*_*)* are stable semiconductors with direct bandgap for visible light absorption, positioning them as a promising candidate for photodetector and optoelectronic applications [43, 82]. Wang et al*.* recently demonstrated the construction of a retinomorphic sensor, where the photoresponses of the sensor are employed to act on a gate terminal within a memristive device, enabling the processing of image data [43]. Furthermore, WSe_2_ exhibits exceptional nonlinear optical response, which can be exploited to realize nonlinear transistors. Tong et al*.* achieved this by integrating WSe_2_ with lithium niobite, realizing nonlinear transistors and non-volatile memory with memory operating functionality [101]. Additionally, Sebastian et al*.* utilized WSe_2_ memtransistors integrated with circuits to implement the hyperbolic tangent and sigmoid activation functions in an artificial neuron [102].

*Tin sulfide (SnS)* is a layered semiconductor with remarkable optical sensitivity, characterized by abundant defect states originating from both Sn and S vacancies, many of which are located within the bandgap. Sun et al*.* recently presented a noteworthy achievement in the fabrication of a SnS-based memristor array designed for language learning, wherein the memristor is directly stimulated by optical signals as the input [81]. The optical and electric stimuli effectively modulate the vacancy states of SnS within the bandgap, taking advantage of the presence of donor and acceptor states in SnS to enable concurrent dual-mode operation for processing optical and electric stimuli.

### Phase Change: MoS_2_

Molybdenum disulfide (MoS_2_) exists in two phases: a semiconducting phase (2H) characterized by a trigonal prismatic polytype atomic configuration, and a metallic phase (1T) with octahedral crystal symmetry atomic configuration. The difference in electronic conductivity between these two phases is harnessed in memristive devices that employ phase transition through the intercalation of Li^+^ ions, as reported by Zhu et al*.* [83]. Figure 2d shows the transition of MoS_2_ from 2H to 1T phases with Li^+^ ions intercalated in between the layers, causing a switch from HRS to LRS. The process involves the drift of Li^+^ ions toward electrodes with lower potential under an external electric field, and the Li^+^ ion accumulation from different terminals, mimicking the cooperative behavior of synapses. In addition, the MoS_2_ is dangling-bond free and highly isotropic, enabling the migration of intercalating ions and their efficient control through electric fields. Hao et al*.* have realized leaky integrate-and-fire neurons based on MoS_2_ through the injection and extraction of Ag^+^ ions under an electric field [103].

### Ferroelectricity: In_2_Se_3_

*α-In*_*2*_*Se*_*3*_ is an anisotropic material with intriguing ferroelectric properties and a direct bandgap of 1.36 eV. Figure 2e shows an In_2_Se_3_-based memtransistor. The origin of ferroelectricity arises from the displacement of Se atom, which switches both in-plane and out-of-plane polarization. An external field is applied to flip the polarization direction. Due to the fading effect of the ferroelectric polarization in α-In_2_Se_3_, volatile α-In_2_Se_3_-based ferroelectric memory has been demonstrated for reservoir computing [84, 104]. In a significant advancement, Liu et al*.* demonstrated an α-In_2_Se_3_-based memristor array for a deep reservoir computing network [85]. The switching behavior of these memristors is achieved through polarization switching, which is effectively controlled by a back gate. This innovative approach holds promise for developing advanced computing systems.

## 2D Material-Based Device Performance Metrics

The concept of in-memory computing draws inspiration from the energy-efficient and collocated data processing and storage characteristics observed in the human brain, which operates at approximately 20 W [7]. In the brain, neural connections are established through synapses, and changes in synaptic plasticity are indicative of memorization and computational processes. Similarly, neuromorphic computing employs artificial synapses and neurons as fundamental components in constructing artificial neural networks.

It is important to note that computing systems integrate diverse computing units to accomplish complex tasks, with each unit responsible for specific functionalities. The performance of such computing systems is influenced by two factors: (1) system-level implementation, which includes the design of the integration framework, integration capability, and density, and (2) the performance of individual computing units. These factors are interdependent, as different integration frameworks impose varying requirements on computing units, thereby calibrating different performance merits for individual devices. For instance, the weight update process in artificial synapses requires minimal cycle-to-cycle variation and high endurance, while the multiply-and-accumulate (MAC) operation in memristive arrays does not necessitate stringent endurance but prioritizes data retention. On the other hand, understanding the strengths and weaknesses of basic computing units enables informed implementations that address limitations, such as employing a one-transistor-one-memristor (1T1R) framework to mitigate sneak path current issues in a memristor crossbar array. Therefore, this review focuses on devices that have demonstrated utility within an array context, while interested readers can refer to existing comprehensive reviews for an in-depth analysis of singular devices [37, 57, 105].

In this section, we discuss the performance requirements of 2D materials-based memristive arrays as the fundamental computing units in hardware implementations of neural networks. We will review device performance in two categories: (1) memristors and memtransistors for the application of artificial synapses and (2) volatile memristors for application of artificial neurons. Additionally, application-dependent device metrics and their impact on various computing tasks in 2D materials-based arrays will be discussed.

### Performance of Memristive Devices as Artificial Synapse

Memristive devices offer a promising approach for emulating artificial synapses, providing a direct mapping between synapse weight and device conductance. The resistance-switching behavior of memristors mimics the updating of synaptic weights during signal transmission. In neural network applications, memristive devices operate in two key scenarios: weight reading and weight updating. In weight reading, the memristive array performs MAC operations, where data multiplication follows Ohm's law and data accumulation is governed by Kirchhoff's law. The conductance of each memristive device must be accurately programmed prior to reading and remain stable throughout the process, emphasizing the crucial requirement of device reliability. Conversely, in weight updating, the synaptic weights need frequent updates based on the gradient of the cost function, demanding endurance and low energy programming during weight updates [106]. Therefore, it is vital to consider various performance characteristics of memristive devices, such as programming energy, program voltage, device area endurance, retention, and device variations, as they play essential roles in different working scenarios. Table 1 summarizes the performance metrics of 2D material-based memristors and memtransistors that have been fabricated with crossbar array structure.

**Table 1:** The performance metrics of 2D material-based memristors and memtransistors that have been fabricated with crossbar array

Functional 2D material	Switching mechanism	Type	Fabrication method	Device dimension (µm × µm)	Array size	Yield (Working device/tested device)	Programming energy	Switching ratio	Cycle-to-Cycle variation (Total cycles)	Device-to-Device variation of SET voltage	Endurance	Retention (s)	Refs
h-BN	Conductive filament formation	Memristor	CVD	3 × 3	10 × 10	98% (102/104)	NA	10^2^ ~ 10^6^	1.53% (120)	5.74% (out of 16 devices)	120 cycles (DC sweep)	10^4^	[55]
h-BN	Conductive filament formation	Memristor	CVD	0.15 × 0.15	10 × 10	NA	20 fJ	~ 500	13.1% (80)	28.9% (out of 6 devices)	8 × 10^4^ (PVS^*^ visible)	Volatile	[55]
h-BN	Conductive filament formation	Memristor	CVD	3 × 3	2 × 1	NA	125 fJ	10^2^	NA	NA	10^2^ cycles (DC sweep)	10^2^	[69]
h-BN	Conductive filament formation	Memristor	CVD	3 × 3 and 0.32 × 0.42	4 × 4 and 100 × 100	Monolayer: 5% (5/100) Multi-layer: 98% (98/100)	NA	~ 10^2^	NA	NA	~ 60 cycles (DC sweep)	NA	[107]
PdSe_x_O_2-x_	Conductive filament formation	Memristor	Exfoliation	3 × 3	3 × 6	100% (18/18)	0.9 pJ	10^3^	3.6% (100)	NA	7 × 10^2^ cycles (DC sweep)	10^5^	[38]
PdSe_2_	Conductive filament formation	Memristor	Exfoliation	3 × 3	5 × 5	100% (25/25)	11.25 pJ	10^2^	~ 6% (100)	NA	10^2^ cycles (DC sweep)	10^5^	[73]
BP	Conductive filament formation	Memristor	Exfoliation	0.1 × 0.1	10 × 10	NA	NA	10^7^	NA	NA	10^2^ cycles (DC sweep)	10^4^	[96]
MoS_2_	Conductive filament formation	Memristor	Printing	0.6 × 0.3	4 × 4	NA	4.5fJ	10^7^	NA	NA	10^2^ cycles (DC sweep)	4 × 10^4^	[34]
MoS_2_	Conductive filament formation	Memristor	CVD	NA	4 × 4	NA	NA	5 ~ 6	NA	NA	5 × 10^2^ cycles (DC sweep)	10^4^	[70]
MoS_2_	Conductive filament formation	Memristor	MBE	5 × 5 and 0.1 × 0.1	10 × 10	NA	140 pJ	~ 160	18.1% (200)	18.5% (out of 73 devices)	10^7^ (PVS blind)	10^5^	[90]
MoS_2_	Conductive filament formation	Memristor	Exfoliation	NA	Synapse 4 × 2 Neurons: 2	NA	65 nJ	10^3^	NA	NA	50 cycles (DC sweep)	NA	[103]
HfSe_2_	Conductive filament formation	Memristor	Solution process	3 × 3	3 × 3	NA	0.8 pJ	~ 150	NA	NA	5 × 10^2^ (PVS visible)	10^4^	[71]
MoS_2_	Vacancy migration	Memtransistor	CVD	length < 1 µm	10 × 10	90% (9/10)	2 pJ to 2 nJ (via gate)	10^3^	NA	NA	~ 8.5 × 10^2^ (DC sweep)	10^5^	[68]
MoS_2_	Vacancy migration	Memtransistor	CVD	0.4 × 20	10 × 10	64% (64/100)	20 fJ	~ 50	5.9% (150)	11.4% (out of 64 devices)	150 cycles (DC sweep)	10^4^	[86]
WS_2_	Photon response	Memtransistor	CVD	1 × 1	6 × 6	NA	14 pJ	50–200	NA	NA	10^2^ cycles (DC sweep)	8 × 10^4^	[82]
SnS	Photon response	Memristor	Exfoliation	length ~ 2 µm	5 × 1	100% (5/5)	NA	NA	NA	NA	NA	NA	[81]
MoS_2_	Phase change	Memristor	Exfoliation	~ 20 × 20	4 × 1	NA	NA	10^2^	NA	NA	4 × 10^4^ (PVS)	7 × 10^3^	[83]
α-In_2_Se_3_	Ferroelectricity	FeFET^*^	Exfoliation	4 × 4	100 × 1	NA	10 fJ	10^6^	NA	NA	10^4^ cycles (DC sweep)	volatile	[84]
α-In_2_Se_3_	Ferroelectricity	FeFET	Exfoliation	NA	2 × 1 × 2(2 layers)	NA	NA	NA	NA	NA	NA	NA	[85]

^*^PVS: pulsed voltage stress          ^*^FeFET: Ferroelectric field-effect transistor

#### Programming Energy

**Fig. 3:**
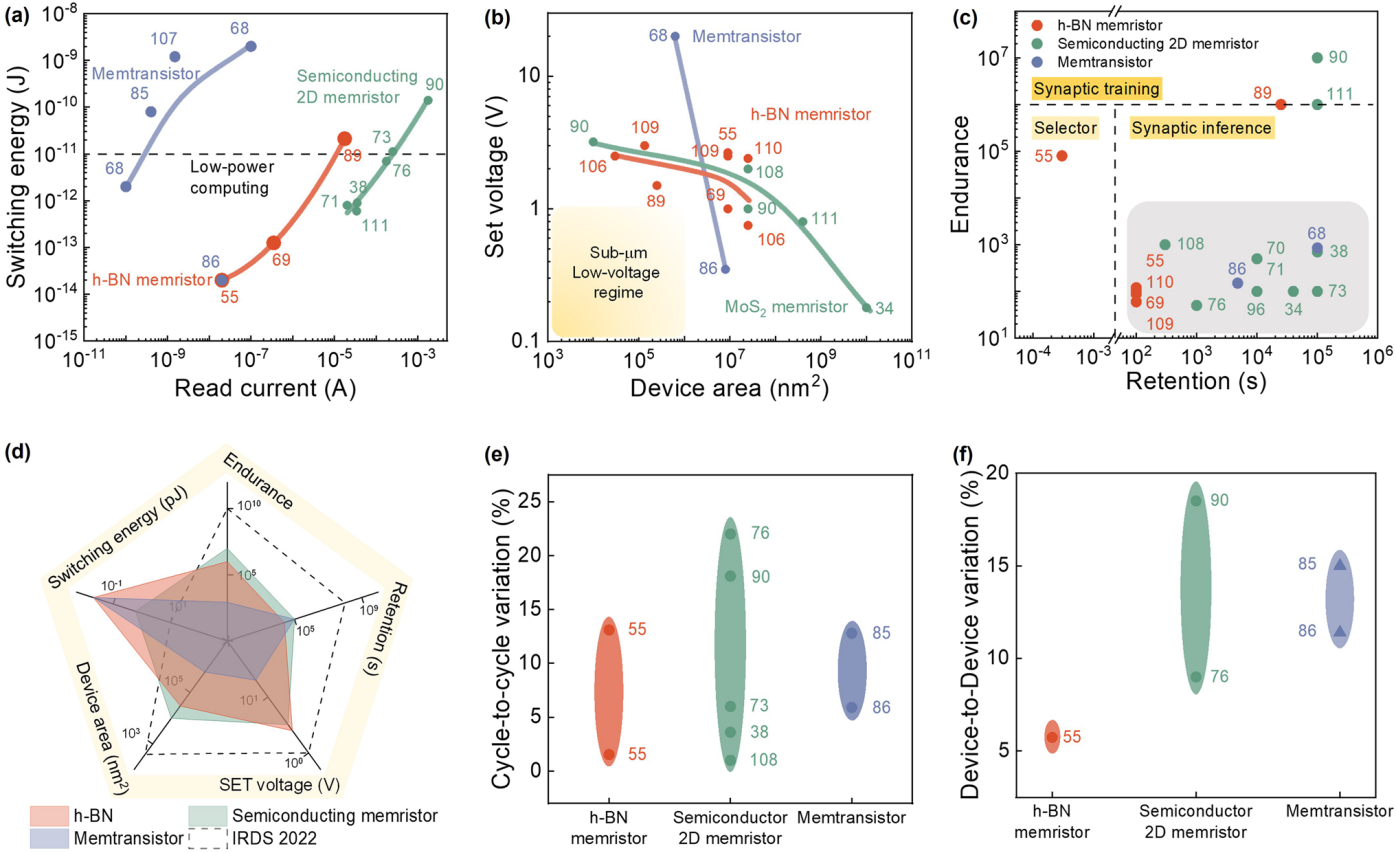
The summary of memristive device performance. **a** Switching energy comparison among insulating 2D h-BN-based memristors, semiconducting 2D material-based memristors, and memtransistors. **b** The relationship between device program voltage and the device size. **c** The reported device endurance and retention and their suitable working scenarios. **d** Radar plot of the key merits of the memristive device and the comparison with International Roadmap for Devices and Systems (IRDS) requirements. **e** Typical cycle-to-cycle variation of 2D material-based memristive devices. **f** Typical device-to-device variation of 2D material-based memristive devices. The numbers in figures correspond to the number in reference list [34, 38, 55, 68–71, 73, 76, 85, 86, 89, 90, 96, 107, 109–113]

The programming energy in memristive devices is a crucial parameter for evaluating energy consumption during weight updates. It is calculated by integrating voltage, current, and time during the device programming process. Figure 3a illustrates the programming energy of various memristive devices implemented in arrays. The results indicate that h-BN-based memristors exhibit the lowest switching energy, operating in the femtojoule range [55, 69]. Even when integrated with CMOS transistors, the energy consumption of h-BN memristors remains within the low-power computing standards proposed by International Roadmap for Devices and Systems (IRDS) [89, 108]. Remarkably, Chen et al*.* suggested that h-BN memristors have the potential to achieve switching energy in the zeptojoule range, approaching the basic thermal energy at room temperature [55]. In contrast, memristors based on other semiconducting 2D materials demonstrate significantly higher switching energy in the order of picojoules [57, 71, 73, 76, 90]. This can be attributed to the unique filament confinement effect within h-BN defects, enabling operation at low compliance currents of 1 μA and lower programming currents of around 100 nA. On the other hand, memtransistors exhibit higher energy consumption despite their lower read currents compared to memristors. This is due to the reliance of memtransistors on vacancy diffusion along grain boundaries, which necessitates strong electric fields and high voltages [44, 77, 80]. Notably, Feng et al*.* have demonstrated ultra-low switching energy in MoS_2_-based memtransistors comparable to h-BN-based memristors, attributed to the high grain boundary density and small grain size within the as-grown MoS_2_ layer, facilitating the migration of sulfur vacancies and reducing energy consumption [86]. In addition to programming energy, the consideration of read energy or compute energy is also crucial as it plays a significant role in synapse inference. The overall energy consumption within an array is determined by the collective read energy of individual devices within the array. Despite its significance, information regarding specific read energy values is limited in the existing literature. Therefore, there is a pressing need to focus research efforts on accurately measuring read energy in order to facilitate the optimization of energy consumption during synapse inference. Overall, h-BN-based memristors show the most potential for low-power computing due to their low read currents and insulating properties. However, further grain boundary engineering is required to improve the energy performance of MoS_2_-based memtransistors. Additionally, defect engineering is necessary for reducing programming currents in other semiconducting 2D material-based memristors.

#### Program Voltage and Device Area

The program voltage is a crucial parameter that exhibits a strong correlation with device size. Figure 3b illustrates the behavior of three kinds of devices: h-BN memristor, MoS_2_ memristor, and memtransistor, all displaying a consistent pattern of increasing set voltage as the device area decreases. This relationship can be attributed to various factors. In the case of memristors, the larger set voltage observed in scaled-down devices stems from the limited availability of defect paths, which necessitates a higher voltage for the formation of conductive filaments [107]. Similarly, for memtransistors, the reduction in device area leads to a decrease in the number of grains between the source and drain terminals, consequently restricting the migration of vacancies [86, 97]. To construct large-scale memristive arrays for hardware neural network applications, achieving high device density and low set voltage at the sub-micrometer range is crucial [114]. However, the survey depicted in Fig. 3b indicates that the state-of-the-art technique has not reached a set voltage of less than 1 V at such small device areas. This highlights the need for further exploration into voltage scaling and device area scaling to meet the requirements of future applications.

#### Endurance and Retention

Endurance refers to the ability of a memristive device to sustain a certain number of operational cycles before its memristive states become unstable and difficult to maintain. On the other hand, retention measures the duration for which memristive states can persist without significant degradation or relaxation. In computation-intensive applications, high endurance is crucial to handle frequent and rapid updates of memristive states required for complex calculations. Conversely, memory devices necessitate high retention to ensure accurate storage of data over extended periods, mitigating the risk of data loss or corruption.

Figure 3c shows the endurance and retention of various types of memristive devices. Tang et al. show that solution-processed MoS_2_-based memristors exhibit high endurance of 10^7^ cycles using voltage pulses [90]. Moreover, its retention is extrapolated to 10 years by showing that the memristive states are not degraded for a duration of 10^5^ s at 85 °C [90]. Similarly, Li et al. observed stable endurance for up to 500 cycles with retention greater than 10^4^ s in a 4 × 4 array of MoS_2_-based memristors [70]. The high endurance and retention of MoS_2_ are also reported in MoS_2_-based memtransistors [68, 110]. Zheng et al*.* conducted tests on a two-state MoS_2_-based memtransistor using 10^9^ voltage pulses without significant changes in its performance [110]. It is interesting to note that black phosphorene-based memristors also show high retention of up to 10^4^ s, primarily due to the layered confinement between phosphorene oxides [96]. In addition, PdSe_2_ devices have shown retention for up to 10^5^ s with 10 stable states maintained at room temperature [73]. Compared to MoS_2_ and other semiconductor-based memristive devices, h-BN-based memristors often exhibit lower endurance and retention. One possible reason for this is that h-BN-based memristors normally have multiple resistance states. Some of the states are unfavorable and may relax to other states once external drift is removed.

Based on the available survey data, most 2D material-based memristive devices and arrays currently exhibit low endurance (< 10^6^ cycles) and acceptable retention (10^4^ ~ 10^5^ s), indicating their potential for demonstrating simple synapse inference where the device retention can sustain synapse weights during computations. However, for synapse training purposes, further efforts are needed to enhance the endurance of 2D material-based memristive devices. Figure 3d presents a radar plot comparing the above-mentioned five parameters with the values proposed by IRDS 2022, revealing that the energy consumption of emerging devices already meets or even exceeds the required performance [108]. However, there is still some disparity between other metrics and the industry's performance expectations.

#### Device-to-Device Variation and Cycle-to-Cycle Variation

Device-to-device variation is a significant constraint in the performance of memristive devices. While individual devices may exhibit excellent performance, the presence of device-to-device variations can hinder their overall performance when integrated into device systems. High device variation not only reduces yield but also significantly impacts the stability of performance in crossbar arrays and other in-memory circuits. Cycle-to-cycle variation is another factor that introduces uncertainty in weight updates, and when integrated into arrays, it further diminishes the precision of weight updates.

Figure 3e, f shows the cycle-to-cycle variation and device-to-device variation of various types of memristive devices, respectively. Notably, memristors based on h-BN show the lowest device-to-device variation, leading to a high yield of 98% [55]. h-BN-based memristors also show low cycle-to-cycle variation. This low variation is primarily due to the overwritten of defects by the most conductive defects, making it less sensitive to defect formation during device fabrication and also during the resistance switching processes.

On the other hand, memristors based on transition metal dichalcogenides (TMDCs) exhibit higher levels of device-to-device and cycle-to-cycle variation. This can be primarily attributed to the uncontrollable defect density during device fabrication and the strong dependence of stochastic filament formation on these defects [71]. Tang et al*.* discussed that the variation in TMDC-based memristors is linked to edge-confined sulfur vacancies, where the size of the MoS_2_ nanosheet affects cycle-to-cycle variation [90]. Specifically, a smaller nanosheet size increases the edge-to-basal plane ratio and reduces cycle-to-cycle variation [90]. In ReS_2_-based memristors, high cycle-to-cycle variation is influenced by Mo-irradiation, which leads to the uncontrolled formation of defects [76]. Moreover, Li et al*.* demonstrated that variation in PdSe_2_ can be improved through electron beam irradiation and oxidation, as the treated material exhibits better control of the filament [38, 73].

MoS_2_-based memtransistors also exhibit higher device variations compared to h-BN-based memristors. This is due to the non-uniform distribution of grain boundaries within the functional layer, resulting in variations in the migration channel. Feng et al*.* suggested reducing the grain size to be smaller than the channel area to mitigate grain boundary variation in each device and subsequently decrease overall variation [86].

It is worth noting that the aforementioned discussions primarily focus on the impact of device variation on synapse inference and synapse training in fully connected artificial neural networks (ANNs). However, in probabilistic systems, such variations serve as a source of randomness and should not be eliminated. For instance, Sebastian et al*.* [102] and Zheng et al*.* [110] utilized the device-to-device variation in MoS_2_-based memtransistors to develop a random number generator and construct a Bayesian network for applications where uncertainty plays a crucial role. Furthermore, Chien et al*.* demonstrated the utilization of cycle-to-cycle variation in MoS_2_ memtransistors for computing security applications [115]. This suggests that while device variation presents challenges, it can also be leveraged to enable novel functionalities and applications.

### Performance of Volatile Memristors as Artificial Neuron

In biological systems, neurons serve as threshold elements, transmitting output signals to postsynaptic neurons once input signals from presynaptic neurons accumulate to a certain threshold. Inspired by biological neuron, artificial neuron has been developed for various applications. For example, Chen et al*.* constructed an ultra-sensitive artificial neuron-like NO_2_ gas-sensing structure based on CuS quantum dots and Bi_2_S_3_ nanosheets [116]. The behavior of neurons can be accurately represented by the leaky integrate-and-fire (LIF) model, where the neuron's input signal is typically a sequence of pulses [117]. The ability of LIF neurons to accumulate stimulation during input pulse trains and recover after pulse trains is crucial. In ANNs, neurons also refer to nonlinear activation functions between weight layers, preventing deep neural networks from reducing to simple linear networks. In this context, the neuron's input is static output values from the previous layer, and achieving high energy efficiency in signal processing is important due to the substantial energy consumption of CMOS-based analog-to-digital converter (ADC) and digital-to-analog converter (DAC) circuits [108].

Artificial neurons can be realized through volatile memristors, which operate based on a threshold switching mechanism. When an input voltage signal is applied to an artificial neuron, it integrates the signal, and upon reaching a threshold voltage, the memristors will switch from HRS to LRS, resulting in the firing of an output current. After firing, the devices revert to their original HRS, owing to their volatile switching property. The time required for such relaxation process corresponds to the refractory period of a neuron.

The performance of artificial neuron is assessed using several criteria. Firstly, a low threshold voltage is essential in order to achieve an energy-efficient artificial neuron. Most threshold switching devices have a threshold voltage in the range of 0.3–1.0 V (as shown in Table 2). Xu et al*.* achieved a remarkably low threshold voltage, approaching 0.1 V, by using MoS_2_ sandwiched between Cu and Au electrodes [41]. This ultra-low threshold voltage is explained by the atomic-scale filament formation combined with electrochemical metallization. Another crucial criterion is the switching ratio, which characterizes the leakage current in the off-state and influences the size of the neuron network. Currently, the switch ratio of threshold switching devices can reach 10^3^ to 10^6^ [103, 118–120]. Another important factor for an artificial neuron is its endurance, which is measured by the number of pulses that the artificial neuron can reliably handle. The reported threshold switching devices are typically tested over tens of pulses. It is worth mentioning that an MoS_2_-based threshold switching memristor is reported to handle up to 10^6^ pulses.

**Table 2:** Performance metrics of 2D material-based artificial neurons based on volatile memristors

Functional 2D material	Type	Fabrication method	Device dimension (µm^2^)	Mechanism	Threshold voltage (V)	Switching ratio	Endurance	Refs
MoS_2_	Memristor	CVD	4	Conductive filamentary formation	0.35 ~ 0.4	10^6^	5 × 10^6^ (PVS)	[117]
MoS_2_	Memristor	CVD	~ 1	Conductive filamentary formation	1.2	10^4^	50 (PVS)	[103]
HfSe_2-x_O_y_	Memristor	Exfoliation	~ 10	Conductive filamentary formation	0.542	10^6^	100 (DC sweep)	[118]
MoS_2_	Memristor	CVD	~ 1	Migration of oxygen ions	NA	~ 10^3^	NA	[119]
MoS_2_	Memristor	CVD	0.01 ~ 1	Conductive filamentary formation	~ 0.1	< 10	40	[120]

It is worth noting that these performance metrics are based on an individual device. However, challenges still remain when integrating them into an artificial neuron network, in which the devices fabricated so far are in the scale of µm^2^, which should be reduced for high-density integration. Recently, Hao et al*.* successfully demonstrated the realization of a network of artificial neurons using 2D planar MoS_2_ [15]. Their devices utilized TiW and Ag electrodes patterned on a single crystal monolayer MoS_2_ to act as the postsynaptic and presynaptic terminals, respectively. The MoS_2_ channel acts as the membrane through which the Ag ions can migrate and diffuse under an electric field, mimicking the injection and extraction of Ca^2+^ ions in a biological neuron. This behavior is facilitated by the high mobility of Ag ions along the MoS_2_ lattice, characterized by a low diffusion barrier of 0.14 eV, resulting in a volatile device. It is important to note that the LIF model is more suitable for spiking neural networks (SNNs), although few demonstrations of artificial neurons for SNNs using 2D materials have been reported, likely due to the complex design of peripheral circuits required to align the firing time with neurons in the previous layer [89].

For nonlinear activation functions, Sebastian et al*.* utilized memtransistor-based integrated circuits to implement hyperbolic tangent and sigmoid activation functions as artificial neurons. By employing memtransistor-based neurons, the Bayesian neural network (BNN) avoids the need for peripheral DAC and ADC components, resulting in reduced energy consumption. Furthermore, these nonlinear functions can also be utilized as hardware reservoirs, sharing the same characteristics as neural activation functions, namely volatility and nonlinearity. For instance, the volatile In_2_Se_3_-based ferroelectric memtransistor can be employed in hardware reservoirs due to the fading effect of the ferroelectric polarization [84].

## Array Configuration and Integration

The performance of a memristive device array is influenced by various factors beyond a simple sum of individual device characteristics. The architectural design of the array also plays a vital role in determining its overall performance. For instance, the power consumption of the array comprises not only the sum of individual device power consumption but also other factors such as energy wasted due to leakage current, wire resistance, and training inefficiency. Therefore, to assess the overall performance of the array, several array-level considerations need to be addressed. These include ensuring the controllability of each device to mitigate leakage current and cross-talk issues, minimizing the array's footprint to enhance integration density and reduce wire resistance, and selecting appropriate connection morphologies to meet diverse application requirements. This section will discuss different memristive array configurations and present typical examples of memristive device integration for high-density crossbar array (CBA) and high energy-efficiency in-memory circuits.

### Passive Memristor CBA

#### One-dimensional (1D) Memristor CBA

**Fig. 4:**
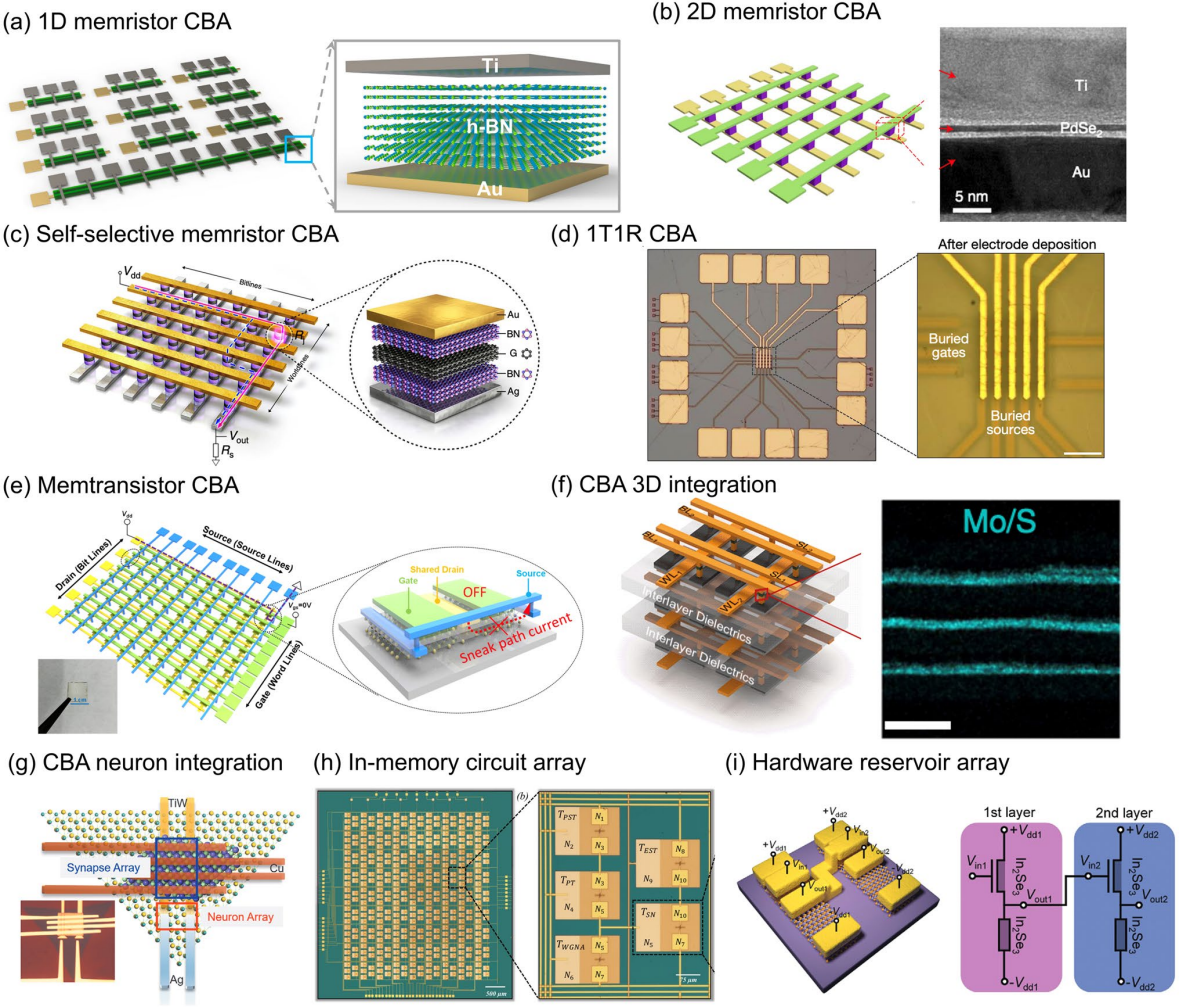
Memristive array configurations and integrated in-memory circuits. **a** 1D memristor passive CBA. **b** 2D memristor passive CBA. **c** Memristor CBA with access selector devices. **d** Memristor CBA with access transistor devices. **e** Self-selective memtransistor CBA. **f** CBA for 3D integration. **g** Integration between synapse CBA and neuron devices. **h** Integrated in-memory circuits and its CBA. **i** Integrated in-memory circuits for multilayer hardware reservoirs. **a** Reproduced from [69]. **b** Reproduced with permission [73], copyright © 2021 Springer Nature Limited. **c** Reproduced from [47]. **d** Reproduced from [89]. **e** Reproduced with permission [86], copyright © 2021, American Chemical Society. **f** Reproduced from [90]. **g** Reproduced with permission [103], copyright © 2020 WILEY‐VCH Verlag GmbH & Co. KGaA, Weinheim. **h** Reproduced from [121]. **i** Reproduced with permission [85], copyright © 2022 Wiley‐VCH GmbH

A 1D memristor array typically consists of multiple word lines (WLs) and one-bit line (BL). It receives vector inputs and generates a single output current. The input is sent to the 1D array through the WLs, and the output is collected at the BL. Due to the single path for current flow in the BL, there is no issue of sneak path leakage current in such an array. Additionally, by applying a voltage to the WLs and grounding the BL, cross-talk issues can be addressed as each WL controls only one device. Consequently, the 1D memristor array is commonly used for basic computing concept verification, as it avoids uncertainties that may arise in large-scale arrays. For example, Xie et al*.* demonstrated a 1D h-BN-based memristor array for multiply-accumulate (MAC) operations, as depicted in Fig. 4a [69]. The output current exhibited distinct variations in response to none-pulsed input, one-pulsed input, and both-pulsed input, indicating the potential for vector–matrix multiplication in synapse inference tasks. Moreover, Sun et al*.* showcased the application of a one-dimensional array for language learning using two-dimensional SnS as the functional material [81]. Optical signals derived from handwritten letters were converted into six digital input signals received by the memristors in a one-dimensional array. The electric response and fading memory of SnS memristors facilitated signal processing through reservoir computing, generating output that could be further classified as vowels and consonants. This one-dimensional array achieved the integration of sensors and processors within a single device. Furthermore, Chien et al*.* demonstrated a 1D synaptic array employing MoS_2_ as a true random number generator for data encryption [115]. Notably, this 1 × 8 array consisted of 1 WL and 8 BLs, enabling the generation of multi-bit random values sequentially by engaging each BL electrode. However, it should be noted that while a 1D array is suitable for simple array-level analysis, its scalability is limited by its dimension, and it is not suitable for parallel computing that is required in modern neural networks.

#### 2D Memristor Passive CBA

The integration of memristive devices can be effectively achieved through the implementation of a crossbar array, as depicted in Fig. 4b. In this configuration, each memristor is positioned at the intersection of a WL and a BL, with the information regarding weights stored in the form of electric resistance within each individual memristor. To update these weights, a voltage pulse is selectively applied between a chosen bit line and word line, thereby initiating the program/erase process. The utilization of such a crossbar array offers several advantages, including a compact footprint that facilitates high-density integration. Furthermore, owing to its straightforward structure and ease of fabrication, it has been widely employed as the predominant architecture for memristor crossbar arrays in various studies [34, 38, 55, 71, 73, 76, 90, 96, 107].

However, it is important to acknowledge that this passive memristor crossbar array is susceptible to challenges related to sneak path current and cross-talk. The sneak path current denotes the unintended flow of current through other memristors when attempting to read a specific memristor, resulting in inaccurate readings and wastage of energy. The cross-talk issue emerges when a selected memristor receives a voltage pulse for weight updates, inadvertently causing adjacent memristors to experience a half-voltage pulse, leading to current leakage and unintended alterations in their respective weights. These issues arise due to the inherent lack of precise control over current flow in this architecture. As a result, this passive crossbar array is most suitable for devices exhibiting high nonlinearities in their voltage-current response, in order to mitigate the occurrence of half-current issues.

### Memristor CBA with Access Selector or Transistor

One approach commonly employed to mitigate the issue of current leakage is the integration of an additional device that handles the selection of memristors during programming and reading operations, such as transistors and selectors. These configurations are typically known as one-selector-one-memristor (1S1R) and one-transistor-one-memristor (1T1R) architectures.

Selectors typically consist of a two-terminal nonlinear device with volatile characteristics. They exhibit high resistance when the voltage is lower than the threshold voltage and low resistance when the voltage exceeds the threshold voltage. This behavior allows them to effectively control the leakage current of half-selected devices. Selectors often share a similar vertical structure as memristors, enabling their integration in a vertical manner to optimize device area utilization. For instance, Sun et al*.* demonstrated a self-selective Au/h-BN/graphene/h-BN/Ag memristor CBA, where the bottom part of the heterostructure (graphene/h-BN/Ag) served as the selector (Fig. 4c) [47]. The CBA achieved exceptional selectivity exceeding 10^10^ with the aid of selective devices.

Transistors are fundamental components with three terminals, while memristors are commonly integrated above the source/drain bias of the underlying transistor. In a 1T1R structure, device selection is achieved by gate voltage at the WLs. Applying varying gate voltages to different WLs allows for low current in unselected devices where the transistor remains off [122, 123]. Furthermore, the devices at the selected WL are further controlled by the BL voltage, which is applied across the channel of the transistor and the memristor. Zhu et al*.* demonstrated a CMOS-based 1T1R crossbar array using h-BN, achieving high endurance and excellent switching uniformity by leveraging the precise compliance current control of the transistor. This work highlights the potential of integrating 2D materials with the Si platform for heterogeneous integration (see Fig. 4d) [89]. Additionally, Yeh et al*.* proposed a novel 0.5T0.5R structure to further downscale the device size of the 1T1R architecture. This structure exploits the edge contact between titanium (Ti) and h-BN to create a memristor at the edge of the underlying transistor [67].

### Multiterminal Memtransistor CBA

In contrast to memristors, memtransistors exhibit additional terminals that enable enhanced control over their switching behavior. The architecture of a memtransistor array is depicted in Fig. 4e, where the functional material (MoS_2_) is interconnected with the source, drain, and gate terminals. Memtransistor crossbar arrays inherently address challenges related to sneak-path current and cross-talk during reading and programming operations by effectively blocking the current flow in unselected memtransistors via their gate terminals. Feng et al*.* successfully fabricated a compact memtransistor crossbar array with a footprint of 3 ~ 4.5 F^2^, surpassing the performance of the 1T1R structure [86]. The memtransistor array demonstrated minimal sneak path leakage current, measuring less than 1 nA. Additionally, apart from the three-terminal memtransistors, dual-gate memtransistors were also developed, incorporating an additional terminal to control device selection within the crossbar array [68]. The sneak path current was checked by measuring the current of a neighboring half-selected device while the selected device underwent four switching cycles. The measurements revealed consistent current values in the half-selected device, indicating the negligible impact of sneak path leakage in such a memtransistor array configuration.

### 3D Integration

Multilayer integration is essential for boosting neuron network performance [124]. The utilization of 2D material-based memristive devices offers a distinct advantage for vertical stacking owing to the stable nature of their layer structures, which are free from dangling bonds and do not encounter lattice mismatch issues. Building upon this concept, Sivan et al*.* proposed a novel 3D stacking architecture for 2D materials between logic and memory units, aiming to enhance integration and performance [56]. However, despite the potential benefits, practical challenges associated with the transfer process of 2D materials continue to hinder the widespread implementation of 3D stacking for memristive devices based on 2D materials. Recently, Tang et al*.* successfully achieved the fabrication of 3D stacked memristors, comprising up to three layers, with each layer measuring approximately 10 nm in thickness [90]. Notably, the solution-processed MoS_2_ was employed in this demonstration, as shown in Fig. 4f. An important aspect of this achievement is the ability to independently program each layer of memristors. The successful demonstration of 3D stacking utilizing memristors based on 2D materials underscores the potential for future integration of high-density 3D structures.

### Integration for In-memory Circuits

#### Synapse CBA Integration with Neurons

In order to achieve the emulation of human brain functionality, the integration of artificial synapses with artificial neurons is crucial to establish functional computing units within a comprehensive neuromorphic network [125]. Hao et al*.* conducted a study where they successfully fabricated a 4 × 2 artificial synapse array, establishing full connectivity with a corresponding array of 2 × 1 neurons (Fig. 4g) [103]. The artificial synapse array was synthesized using Cu/GeTe, while the artificial neuron was synthesized utilizing MoS_2_. By programming the weights based on offline training mapping, the resulting synapse-neuron device array exhibited the desired integration-and-fire behavior. This experimental work serves as a significant demonstration of the potential for integrating synapses and neurons to realize functional behavior in neuromorphic systems.

#### In-memory Circuit Design

In addition to standalone crossbar arrays of memristors/memtransistors, the integration of memristive devices with other circuit elements, such as capacitors and diodes, offers significant potential for achieving specialized functionalities. For instance, Dodda et al*.* conducted research on an array of hybrid devices capable of sensing, encoding, and decoding images [121]. Their device configuration involved an 8 × 8 crossbar array (shown in Fig. 4h) consisting of MoS_2_-based memtransistors, complemented by circuits serving as a Gaussian noise adder, information encoder, and decoder, thereby enabling multifunctionality within the array. Tong et al*.* demonstrated the amplification and operational memory capabilities of a cascaded architecture comprising tungsten diselenide (WSe_2_) and lithium niobite (LiNbO_3_), suitable for binary classification tasks [101]. Additionally, the integration of in-memory circuits lends itself well to the implementation of hardware reservoir computing. For example, Liu et al*.* successfully demonstrated a two-layer reservoir utilizing synaptic transistors based on In_2_Se_3_ [85]. In this configuration, the output (*V*_out1_ in Fig. 4i) from the first layer's nonlinear response was further transmitted to the second layer for additional encoding of the input data (*V*_in1_) into a nonlinear output (*V*_out2_).

## Functionality and Performance Evaluation

In-memory computing has gained significant attention due to its diverse applications in image classification, language recognition, and the internet of things. To enable these applications, the integration of memristive devices is crucial for the development of functional hardware systems. Neuron network, drawing inspiration from the human brain, exhibits wide applications such as sound identification [126], health monitoring [127], language transition [128]. Neural networks also serve as a common approach to integrating memristive devices for neuromorphic computing and artificial intelligence and demonstrate strong performance in many areas such as vision sensor [129], pressure sensor [130], flexible devices [131]. In the existing literature, several types of ANNs have been extensively studied, including fully connected ANNs, SNNs, recurrent neural networks (RNNs), BNNs, and convolutional neuron networks (CNNs). Despite the variations in these network models, they all rely on fundamental functionalities provided by memristive devices. These functionalities encompass essential operations such as weight storage for memorization, matrix–vector multiplication and accumulation (MAC) for computation, linear regression for loss minimization, logistic regression with sigmoid activation for nonlinearity, and convolution image processing with programmable kernels.

While a fully hardware-based implementation of a functional neural network solely relying on memristive devices without digital computing support has not yet been achieved in the current state-of-the-art literature, significant progress has been made in realizing these fundamental functionalities. Consequently, this section aims to comprehensively review the achievements in utilizing 2D materials to realize these basic functionalities. Additionally, possible implementations of various artificial neural networks and their corresponding applications will be discussed, highlighting the potential of memristive devices in advancing the field of in-memory computing and neural network hardware.

### Basic Functionalities Based on 2D Materials

#### Pattern Memorization

**Fig. 5:**
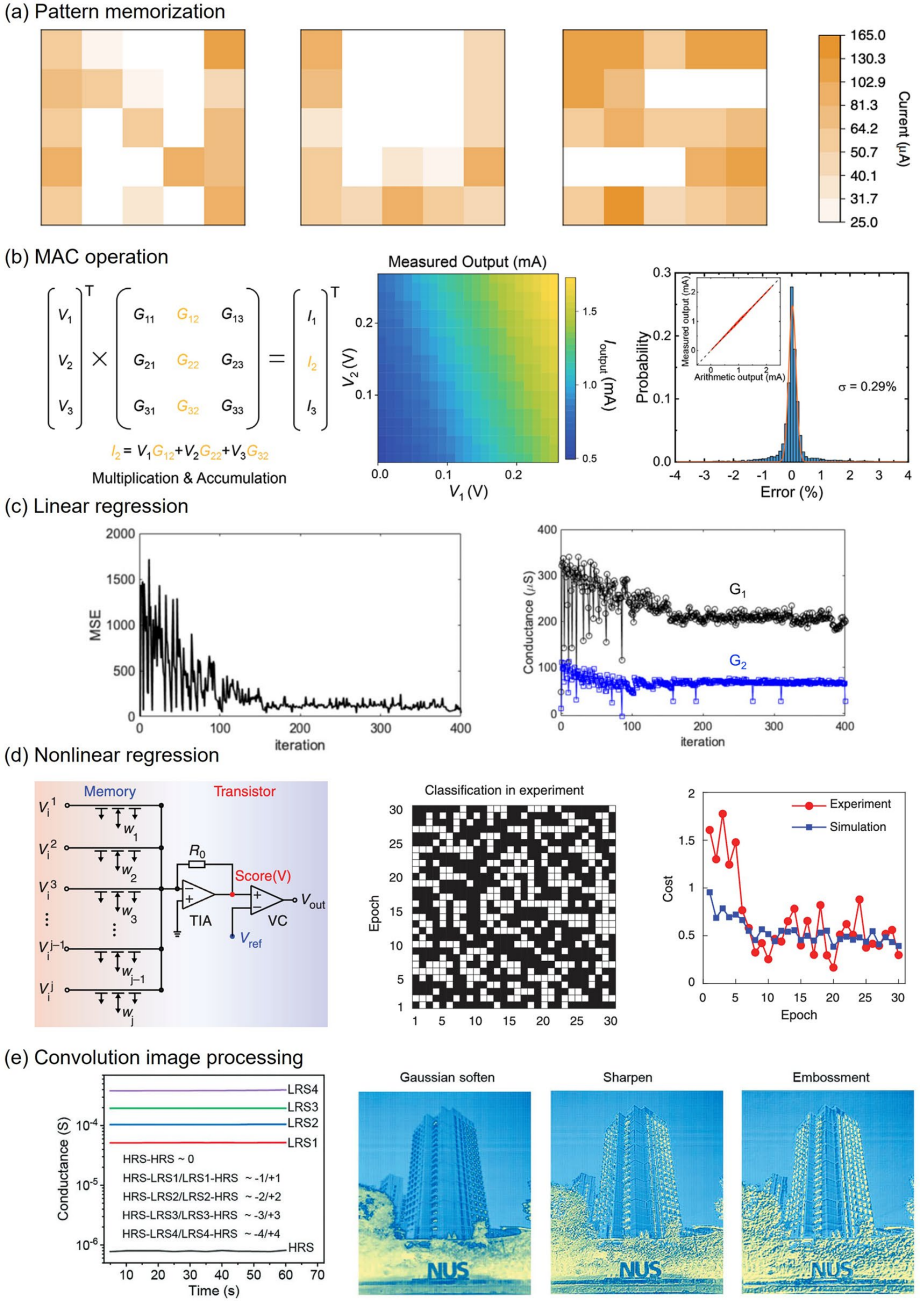
Fundamental functionalities of memristive arrays. **a** Pattern memorization based on a 5 × 5 PdSe_2_ memristor array. **b** MAC operation based on a 3 × 3 HfSe_2_ memristor array. **c** Linear regression using a 2 × 1 h-BN array. **d** Nonlinear regression with activation function using WSe_2_ synaptic transistors and activation circuits. **e** Convolution image processing using a 6 × 3 PdSe_2_ memristor array. **a** Reproduced with permission [73], copyright © 2021 Springer Nature Limited. **b** Reproduced with permission [71], copyright © 2021 Wiley‐VCH GmbH. **c** Reproduced from [69]. **d** Reproduced with permission [101], copyright © 2021, The American Association for the Advancement of Science. **e** Reproduced from [38]

Memorization is a fundamental capability exhibited by memristive devices, akin to the functioning of the human brain, where it can be categorized into two forms: short-term memory (STM) and long-term memory (LTM). The successful realization of STM and LTM relies on achieving a delicate balance between retaining and relaxing resistive states, corresponding to the preservation and fading of stored information. Li et al*.* conducted an experimental study utilizing electron-beam-irradiated PdSe_2_ as the functional material, demonstrating the coexistence of these memory types within a single memristor [73]. In their investigation, a 5 × 5 crossbar array exhibited memorized patterns resembling the letters "N", "U" and "S" as depicted in Fig. 5a. The findings revealed that when subjected to a small pulse (0.9 V), the memorization gradually diminished over one day. Conversely, an application of a larger pulse (2 V) resulted in sustained memorization, effectively transitioning from STM to LTM. This ability to memorize multiple patterns and the selectivity observed in CBA devices holds crucial role for weight storage in neural networks. The demonstrated capability of multipattern memorization highlights the immense potential of memristive devices in emulating complex neuromorphic behaviors and facilitating in-memory computing.

#### MAC Operation

Performing matrix multiplication and accumulation (MAC) operations is a computationally intensive task in traditional digital computers, resulting in substantial energy consumption due to frequent data transfers between memory and processing units. However, utilizing a crossbar array offers a lightweight approach to executing MAC operations. In this approach, the weights representing matrix elements are programmed into memristors, while the input vector is converted into voltage signals applied to WLs. The resulting output currents from BLs represent the MAC result, following the principles of Ohm's law (multiplication) and Kirchhoff's current law (accumulation). This computation exhibits massive parallelism and high energy efficiency in an ideal scenario.

Nevertheless, achieving high accuracy in MAC operations poses challenges due to variations among memristor devices and discrepancies in analog-to-digital conversion. Therefore, ongoing research efforts are focused on enhancing the accuracy of these operations. Li et al*.* conducted an analysis of error probabilities in MAC operations using a HfSe_2_-based memristor array, as illustrated in Fig. 5b [71]. They achieved a small error with a standard deviation of 0.29%, which is sufficiently low for most machine-learning applications. This indicates that a memristive crossbar array can fulfill the accuracy requirements for neuromorphic learning by effectively managing device variations.

#### Linear Regression

Linear regression plays a crucial role in the optimization process during the training of artificial neural networks, aiming to minimize overall loss by updating the weights. The effectiveness of training and the accuracy of predictions heavily rely on the performance of linear regression. In a study conducted by Xie et al., the implementation of multivariable linear regression was demonstrated using a 2 × 1 crossbar array of h-BN memristors [69]. However, due to challenges in hardware implementation, this particular linear regression approach utilized a single programming pulse of fixed width for weight updates. The performance of this approach, as illustrated in Fig. 5c, indicates successful loss minimization. It should be noted, though, that the current parameter size for linear regression is relatively small, representing an initial attempt at hardware implementation of the optimization process in neuromorphic computing. Further advancements in linear regression within larger-scale crossbar arrays are necessary to enable full hardware neural networks.

#### Nonlinear Regression

The activation function plays a crucial role in introducing nonlinearity within neural networks, preventing the regression task from reducing to a simple linear regression. Similar to the human brain, where neurons exhibit nonlinearity in signal processing, the leaky integrate-and-fire (LIF) model has been widely adopted to emulate neuronal functionality [103]. Within this model, neurons receive input signals from synapses, and if the accumulated strength of these signals surpasses a certain threshold, the neuron generates an output signal that propagates through the axon to the next neuron. This process modulates synaptic plasticity, leading to spike-dependent plasticity. Activation functions, such as the commonly used sigmoid function, are mathematically employed to capture the nonlinear behavior of neurons in digital machine-learning models. In hardware-based neuromorphic computing, these activation functions can be realized through external circuits like voltage comparators or through memristive devices such as MoS_2_-based artificial neurons [102]. Figure 5d illustrates the nonlinear response of a voltage comparator, closely resembling the sigmoid function [101]. By integrating activation functions, logistic regression can be performed in hardware networks and the reduction of cost function with training epoch can be achieved, demonstrating the potential of employing 2D material-based deep neural networks for various applications.

#### Image Processing

Image processing encompasses various techniques aimed at extracting relevant information or features from an image, including filtering and segmentation. In the context of convolutional image processing, a kernel is employed to extract specific features, such as edges or patterns, from the image. As illustrated in Fig. 5e, an example of convolution image processing utilizing a 3 × 3 kernel is presented [38]. The kernel values are encoded into a CBA using the column differential method, wherein the difference in conductance between a pair of memristors represents the kernel values. Remarkably, Li et al*.* achieved the programming of kernels with multiple bits using a PdSeO_x_/PdSe_2_ CBA, resulting in convolution kernels endowed with multiple bits and realistic functionalities, including Gaussian softening, sharpening, and embossing. It is worth noting that such convolution operations hold crucial significance in CNN applications, wherein these operations serve to extract image features and transmit them to subsequent layers.

### System-Level Implementation of Neural Network Applications

**Fig. 6:**
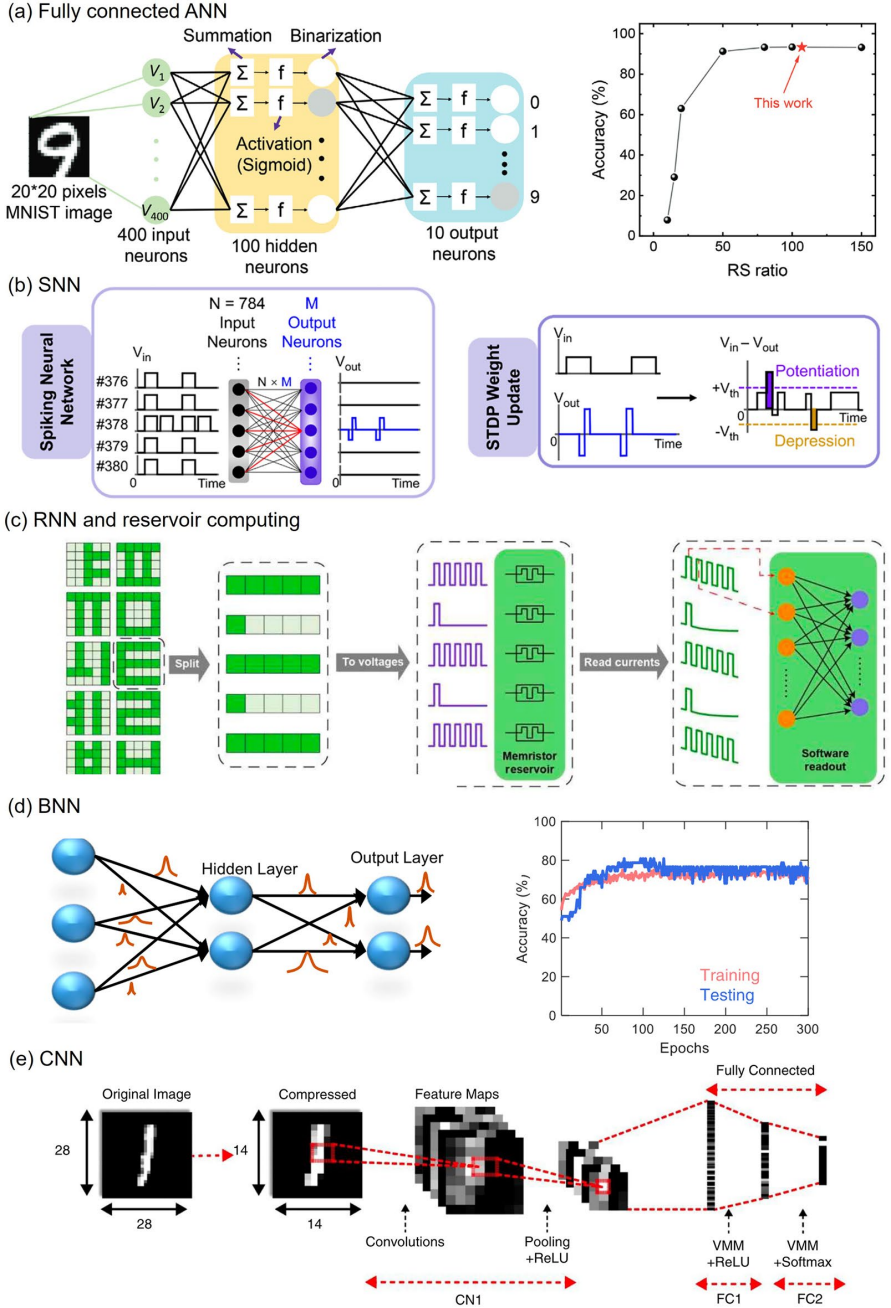
System-level implementation for neural network applications. **a** Fully connected neural networks for MNIST dataset pattern recognition using HfSe_2_-based memristors. The right panel shows the relationship between offline classification accuracy and the HfSe_2_-based memristor RS ratio. **b** SNN for MNIST pattern recognition using MoS_2_-based memtransistors. **c** RNN and reservoir computing using SnS-based memristor for language recognition. **d** BNN for prediction of PIMA diabetes dataset using MoS_2_-based memtransistors. **e** CNN for MNIST dataset pattern recognition using MoS_2_-based memristors. **a** Reproduced with permission [71], copyright © 2021 Wiley‐VCH GmbH. **b** Reproduced with permission [100], copyright © 2021, American Chemical Society. **c** Reproduced from [81]. **d** Reproduced from [102]. **e** Reproduced from [90]

ANNs are computational models that integrate fundamental functions to facilitate training, learning, and prediction. ANNs consist of interconnected artificial neurons that receive input signals as applied voltage, process the signals in a crossbar array, and generate output signals as electric currents. Signal processing involves linear transformations facilitated by MAC operations and nonlinear modifications enabled by artificial neurons [38, 68, 71, 103]. The architecture of a crossbar array represents the interconnected nodes in an ANN model. Currently, most implementations employ offline classification, where trained weights are programmed into memristive devices within the crossbar array [71, 86]. For offline classification, the RS ratio is crucial to the recognition accuracy. As shown in Fig. 6a, the high on/off ratio around 100 times can achieve high recognition accuracy of 93.34% [71].

SNNs closely mimic the operation of biological neurons. In SNNs, input and output signals are represented as spiking pulses, and information is encoded in the timing of these pulses [100, 112]. The transmission of these pulses modifies the connections between neurons, known as synaptic plasticity. SNNs find applications in areas such as time-series analysis and sensory processing (Fig. 6b) [89].

RNNs are designed to simulate dynamic systems like natural language processing or speech recognition (Fig. 6c) [81]. Reservoir computing is a typical example of RNN, and input signals are fed into reservoirs with complex structures, acting as black boxes that process the inputs. The readout mechanism is then trained to map desired outputs to the inputs. The dynamic nature of RNNs requires functional materials to exhibit volatile behavior to represent fading and retention of memory, enabling the simulation of real-time processes [81, 84, 85].

BNNs utilize random numbers as weights instead of constant values. In BNNs, each weight in the neural network is assigned a random number following a Gaussian distribution (Fig. 6d). This approach enables BNNs to handle problems with uncertainties and provide results in the form of probabilities. Neuromorphic devices can exploit the variation of memristive devices to generate random numbers [102, 110], allowing the construction of robust and fault-tolerant neuromorphic systems that are less sensitive to device-to-device and cycle-to-cycle variations.

CNNs are designed to perform feature extraction prior to classification (Fig. 6e). CNNs utilize convolutional layers to detect local features, which are then combined in subsequent layers to recognize global features. In neuromorphic computing, memristor arrays can be employed to realize such CNN architectures [90].

## Current Challenges and Future Outlook

Memristors and memtransistors based on 2D materials have emerged as a rapidly advancing field with substantial potential for enabling neuromorphic computing and in-memory computing. Extensive research efforts have led to the synthesis of memristors and memtransistors with remarkable performance, showcasing significant improvements in recent years [132]. As the field progresses, the synthesis of memristive device arrays has become a focal point in recent literature, representing a critical milestone toward the realization of future neuromorphic computing systems. However, the successful fabrication of high-performance memristive arrays presents considerable challenges that demand attention.

A primary challenge lies in the downscaling of memristors and memtransistors. Device scaling can be divided into two aspects: reducing the thickness of 2D materials and scaling down the device's features. In the case of memristor-based CBA, reducing thickness is crucial as it can lower the switching voltage of memristors, thereby reducing energy consumption in CBAs for low-power in-memory computing applications. Nonetheless, the ultra-thin nature of 2D materials imposes stringent demands on both the growth and integration techniques of these materials. Regarding growth methods, thinner 2D material films exhibit greater thickness variation, resulting in larger variations in switching voltage for memristors. As for integration techniques, thinner layers are more vulnerable to wrinkles and microscopic holes. These wrinkles can significantly alter the effective thickness of the switching medium in thin 2D layers, leading to substantial variations during the electroforming step. Furthermore, if the memristor switching medium contains microscale holes, the device will short-circuit, rendering it incapable of further resistive switching and resulting in low yield.

Scaling down the device feature size is also crucial for both 2D material-based memristors and memtransistors. This enables the construction of large-scale, high-density memristor CBAs, which are essential for complex neural networks used in real-world applications like VGG and YOLO [133, 134]. However, as depicted in Fig. 3b, reducing the size of memristors and memtransistors leads to an increase in the operating voltage of these devices due to the absence of defect paths or grain boundaries in smaller areas. The absence of 2D material-based memristors and memtransistors with sub-micrometer dimensions and sub-1 V switching voltages highlights the disparity between these devices and the integration requirements of advanced CMOS technology. In summary, the challenges in scaling down 2D material-based memristors and memtransistors predominantly stem from issues related to low yield, significant variations in operating voltage, and the escalation of switching voltage and energy during the scaling process. Addressing these challenges necessitates further technological development, with a focus on 2D material synthesis, integration processes, and defect engineering to enable controlled conductive pathways in small areas.

Another significant challenge in 2D material-based memristor technology is the integration of memristors with access selectors or transistors to create large-scale crossbar arrays. Despite the successful demonstration of high device selectivity in 2D material-based 1S1R and 1T1R CBAs [47, 89, 135], they still face challenges related to operating voltage, device latency, and footprint. Firstly, when selectors and transistors are turned on, they act as resistors, necessitating an increase in voltage across the 1T1R and 1S1R cells to achieve the same switching voltage as the 1R memristor cell. While some memristors exhibit a low switching voltage of less than 1 V, they can show significantly higher switching voltages (3 ~ 5 V) after integration [47, 55, 72, 89]. This increase in switching voltage poses a challenge in reducing the energy consumption of the integrated device. Furthermore, the forming voltage of 2D material-based memristors typically exceeds 1 V, which may surpass the voltage supply tolerance of advanced CMOS transistors, thereby limiting the functionality of access transistors. Secondly, device switching speed can be impeded by the latency in selectors and transistors. For instance, in the case of state-of-the-art CMOS/h-BN heterogeneous integration 1T1R CBAs, a high latency of 230 μs during programming is demonstrated, which is considerably slower than memristor-only devices (10 ns) [89]. Moreover, the disparity between the reset current of 2D memristors (1 mA) and the ON-state current of selectors/transistors (100 μA) necessitates a larger area for the selector and transistor arrays to provide sufficient current for switching 2D material-based memristors [34, 36, 55, 71, 73, 136]. In summary, further investigation is warranted for selectors and transistors with a high ON-current, high ON/OFF ratio, and low latency. Additionally, research efforts should be directed towards 2D material-based memristors exhibiting forming-free behavior and reduced reset currents.

Another key challenge involves ensuring the controllability of device variation and switching mechanisms within the arrays. Achieving a high level of controllability is essential to address this issue, as device variation directly impacts yield and, consequently, the cost-effectiveness of the devices. Notably, the fabrication of highly uniform characteristics in TMDC-based memristors and memtransistors remains challenging. Lack of precise control methods over programming voltage and *I–V* characteristics further exacerbates this problem. In addition, quantitative models for some switching behaviors are still unclear, including the low retention time of memtransistors and the high forming voltage of the memristors. To tackle these challenges, a promising approach involves integrating experimental work with theoretical investigations and simulations to identify key factors contributing to device variations and develop strategies to control them effectively [97].

Furthermore, synapse training poses a significant challenge in current technology. Synapse training is crucial for enabling systems to learn and adapt, thereby broadening the potential applications of neuromorphic computing. However, current applications are predominantly limited to synapse inference and software training, and full-hardware online training attempts have been scarce. The primary obstacle lies in achieving high accuracy with minimal programming errors, which is essential for generating effective training. The existing CBAs devices have not yet met the stringent standards required for online training, as programming errors can arise from cycle-to-cycle variation, discrepancies in writing and reading margins among devices, and endurance limitations of certain devices. Consequently, the feasibility of online training remains constrained.

In conclusion, the considerable potential of memristor and memtransistor arrays for enabling neuromorphic computing has been demonstrated and recent years have witnessed remarkable progress and rapid advancements toward next-generation neuromorphic computing systems. Building upon this momentum, it is believed that memristive devices based on 2D materials are poised to shape the future of next-generation in-memory computing systems.

## References

[1] W. Zhang, B. Gao, J. Tang, P. Yao, S. Yu et al., Neuro-inspired computing chips. Nat. Electron. **3**, 371–382 (2020). 10.1038/s41928-020-0435-7

[2] D. Kuzum, S. Yu, H.-S. Philip Wong, Synaptic electronics: materials, devices and applications. Nanotechnology **24**, 382001 (2013). 10.1088/0957-4484/24/38/382001 23999572 10.1088/0957-4484/24/38/382001

[3] D. Li, X. Liang, Neurons mimicked by electronics. Nature **554**, 472–473 (2018). 10.1038/d41586-018-02025-x 10.1038/d41586-018-02025-x 32094933

[4] G. Indiveri, B. Linares-Barranco, R. Legenstein, G. Deligeorgis, T. Prodromakis, Integration of nanoscale memristor synapses in neuromorphic computing architectures. Nanotechnology **24**, 384010 (2013). 10.1088/0957-4484/24/38/384010 23999381 10.1088/0957-4484/24/38/384010

[5] V.K. Sangwan, M.C. Hersam, Neuromorphic nanoelectronic materials. Nat. Nanotechnol. **15**, 517–528 (2020). 10.1038/s41565-020-0647-z 32123381 10.1038/s41565-020-0647-z

[6] Y.-C. Chen, C.-Y. Lin, H. Cho, S. Kim, B. Fowler et al., Current-sweep operation on nonlinear selectorless RRAM for multilevel cell applications. J. Electron. Mater. **49**, 3499–3503 (2020). 10.1007/s11664-020-07987-1

[7] Q. Xia, J.J. Yang, Memristive crossbar arrays for brain-inspired computing. Nat. Mater. **18**, 309–323 (2019). 10.1038/s41563-019-0291-x 30894760 10.1038/s41563-019-0291-x

[8] S. Yu, Neuro-inspired computing with emerging nonvolatile memorys. Proc. IEEE **106**, 260 (2018). 10.1109/JPROC.2018.2790840

[9] A. Sebastian, M. Le Gallo, R. Khaddam-Aljameh, E. Eleftheriou, Memory devices and applications for in-memory computing. Nat. Nanotechnol. **15**, 529–544 (2020). 10.1038/s41565-020-0655-z 32231270 10.1038/s41565-020-0655-z

[10] E.J. Fuller, S.T. Keene, A. Melianas, Z. Wang, S. Agarwal et al., Parallel programming of an ionic floating-gate memory array for scalable neuromorphic computing. Science **364**, 570–574 (2019). 10.1126/science.aaw5581 31023890 10.1126/science.aaw5581

[11] J.J. Yang, D.B. Strukov, D.R. Stewart, Memristive devices for computing. Nat. Nanotechnol. **8**, 13–24 (2013). 10.1038/nnano.2012.240 23269430 10.1038/nnano.2012.240

[12] M. Prezioso, M.R. Mahmoodi, F.M. Bayat, H. Nili, H. Kim et al., Spike-timing-dependent plasticity learning of coincidence detection with passively integrated memristive circuits. Nat. Commun. **9**, 5311 (2018). 10.1038/s41467-018-07757-y 30552327 10.1038/s41467-018-07757-y PMC6294012

[13] C. Li, M. Hu, Y. Li, H. Jiang, N. Ge et al., Analogue signal and image processing with large memristor crossbars. Nat. Electron. **1**, 52–59 (2018). 10.1038/s41928-017-0002-z

[14] M. Hu, C.E. Graves, C. Li, Y. Li, N. Ge et al., Memristor-based analog computation and neural network classification with a dot product engine. Adv. Mater. **30**, 1705914 (2018). 10.1002/adma.201705914 10.1002/adma.201705914 29318659

[15] Y. Li, K.-W. Ang, Hardware implementation of neuromorphic computing using large-scale memristor crossbar arrays. Adv. Intell. Syst. **3**, 2000137 (2021). 10.1002/aisy.202000137

[16] M.A. Lastras-Montaño, K.-T. Cheng, Resistive random-access memory based on ratioed memristors. Nat. Electron. **1**, 466–472 (2018). 10.1038/s41928-018-0115-z

[17] A. Thomas, Memristor-based neural networks. J. Phys D-Appl. Phys. **46**, 093001 (2013). 10.1088/0022-3727/46/9/093001

[18] L. Chua, Resistance switching memories are memristors. Appl. Phys. A **102**, 765–783 (2011). 10.1007/s00339-011-6264-9

[19] Y. Xi, B. Gao, J. Tang, A. Chen, M.-F. Chang et al., In-memory learning with analog resistive switching memory: a review and perspective. Proc. IEEE **109**, 14–42 (2021). 10.1109/JPROC.2020.3004543

[20] D.B. Strukov, G.S. Snider, D.R. Stewart, R.S. Williams, The missing memristor found. Nature **453**, 80–83 (2008). 10.1038/nature06932 18451858 10.1038/nature06932

[21] L. Chua, Memristor-the missing circuit element. IEEE Trans. Circuit Theory **18**, 507–519 (1971). 10.1109/TCT.1971.1083337

[22] H.-S.P. Wong, H.-Y. Lee, S. Yu, Y.-S. Chen, Y. Wu et al., Metal–oxide RRAM. Proc. IEEE **100**, 1951–1970 (2012). 10.1109/JPROC.2012.2190369

[23] R. Waser, R. Dittmann, G. Staikov, K. Szot, Redox-based resistive switching memories: nanoionic mechanisms, prospects, and challenges. Adv. Mater. **21**, 2632–2663 (2009). 10.1002/adma.200900375 36751064 10.1002/adma.200900375

[24] G.W. Burr, R.M. Shelby, S. Sidler, C. di Nolfo, J. Jang et al., Experimental demonstration and tolerancing of a large-scale neural network (165 000 synapses) using phase-change memory as the synaptic weight element. IEEE Trans. Electron Dev. **62**, 3498–3507 (2015). 10.1109/TED.2015.2439635

[25] V. Joshi, M. Le Gallo, S. Haefeli, I. Boybat, S.R. Nandakumar et al., Accurate deep neural network inference using computational phase-change memory. Nat. Commun. **11**, 2473 (2020). 10.1038/s41467-020-16108-9 32424184 10.1038/s41467-020-16108-9 PMC7235046

[26] S. Choi, S.H. Tan, Z. Li, Y. Kim, C. Choi et al., *SiGe* epitaxial memory for neuromorphic computing with reproducible high performance based on engineered dislocations. Nat. Mater. **17**, 335–340 (2018). 10.1038/s41563-017-0001-5 29358642 10.1038/s41563-017-0001-5

[27] J. Lee, C. Du, K. Sun, E. Kioupakis, W.D. Lu, Tuning ionic transport in memristive devices by graphene with engineered nanopores. ACS Nano **10**, 3571–3579 (2016). 10.1021/acsnano.5b07943 26954948 10.1021/acsnano.5b07943

[28] J.H. Yoon, J.H. Han, J.S. Jung, W. Jeon, G.H. Kim et al., Highly improved uniformity in the resistive switching parameters of TiO_2_ thin films by inserting Ru nanodots. Adv. Mater. **25**, 1987–1992 (2013). 10.1002/adma.201204572 23386379 10.1002/adma.201204572

[29] W.-Y. Chang, C.-A. Lin, J.-H. He, T.-B. Wu, Resistive switching behaviors of ZnO nanorod layers. Appl. Phys. Lett. **96**, 242109 (2010). 10.1063/1.3453450

[30] H.-P. Wong, S. Raoux, S. Kim, J. Liang, J.P. Reifenberg et al., Phase change memory. Proc. IEEE **98**, 2201 (2010). 10.1109/JPROC.2010.2070050

[31] C. Liu, H. Chen, S. Wang, Q. Liu, Y.G. Jiang et al., Two-dimensional materials for next-generation computing technologies. Nat. Nanotechnol. **15**, 545–557 (2020). 10.1038/s41565-020-0724-3 32647168 10.1038/s41565-020-0724-3

[32] F. Zhang, H. Zhang, S. Krylyuk, C.A. Milligan, Y. Zhu et al., Electric-field induced structural transition in vertical MoTe_2_- and Mo_1-__*x*_W_*x*_Te_2_-based resistive memories. Nat. Mater. **18**, 55–61 (2019). 10.1038/s41563-018-0234-y 30542093 10.1038/s41563-018-0234-y

[33] A.A. Bessonov, M.N. Kirikova, D.I. Petukhov, M. Allen, T. Ryhänen et al., Layered memristive and memcapacitive switches for printable electronics. Nat. Mater. **14**, 199–204 (2015). 10.1038/nmat4135 25384168 10.1038/nmat4135

[34] X. Feng, Y. Li, L. Wang, S. Chen, Z.G. Yu et al., Neuromorphic computing: a fully printed flexible MoS_2_ memristive artificial synapse with femtojoule switching energy. Adv. Electron. Mater. **5**, 1970061 (2019). 10.1002/aelm.201970061

[35] P. Cheng, K. Sun, Y.H. Hu, Memristive behavior and ideal memristor of 1T phase MoS_2_ nanosheets. Nano Lett. **16**, 572–576 (2016). 10.1021/acs.nanolett.5b04260 26654683 10.1021/acs.nanolett.5b04260

[36] W. Huh, S. Jang, J.Y. Lee, D. Lee, D. Lee et al., Synaptic barristor based on phase-engineered 2D heterostructures. Adv. Mater. **30**, e1801447 (2018). 10.1002/adma.201801447 30015988 10.1002/adma.201801447

[37] C. Zhang, H. Zhou, S. Chen, G. Zhang, Z.G. Yu et al., Recent progress on 2D materials-based artificial synapses. Crit. Rev. Solid State Mater. Sci. **47**, 665–690 (2022). 10.1080/10408436.2021.1935212

[38] Y. Li, S. Chen, Z. Yu, S. Li, Y. Xiong et al., In-memory computing using memristor arrays with ultrathin 2D PdSeO_*x*_/PdSe_2_ heterostructure. Adv. Mater. **34**, e2201488 (2022). 10.1002/adma.202201488 35393702 10.1002/adma.202201488

[39] H. Zhao, Z. Dong, H. Tian, D. DiMarzi, M.-G. Han et al., Atomically thin femtojoule memristive device. Adv. Mater. **29**, 1703232 (2017). 10.1002/adma.201703232 10.1002/adma.201703232 29067743

[40] R. Ge, X. Wu, M. Kim, J. Shi, S. Sonde et al., Atomristor: nonvolatile resistance switching in atomic sheets of transition metal dichalcogenides. Nano Lett. **18**, 434–441 (2018). 10.1021/acs.nanolett.7b04342 29236504 10.1021/acs.nanolett.7b04342

[41] R. Xu, H. Jang, M.-H. Lee, D. Amanov, Y. Cho et al., Vertical MoS_2_ double-layer memristor with electrochemical metallization as an atomic-scale synapse with switching thresholds approaching 100 mV. Nano Lett. **19**, 2411–2417 (2019). 10.1021/acs.nanolett.8b05140 30896171 10.1021/acs.nanolett.8b05140

[42] X. Wu, R. Ge, P.-A. Chen, H. Chou, Z. Zhang et al., Thinnest nonvolatile memory based on monolayer h-BN. Adv. Mater. **31**, e1806790 (2019). 10.1002/adma.201806790 30773734 10.1002/adma.201806790

[43] S. Wang, C.-Y. Wang, P. Wang, C. Wang, Z.-A. Li et al., Networking retinomorphic sensor with memristive crossbar for brain-inspired visual perception. Natl. Sci. Rev. **8**, nwaa172 (2020). 10.1093/nsr/nwaa172 34691573 10.1093/nsr/nwaa172 PMC8288371

[44] V.K. Sangwan, H.-S. Lee, H. Bergeron, I. Balla, M.E. Beck et al., Multi-terminal memtransistors from polycrystalline monolayer molybdenum disulfide. Nature **554**, 500–504 (2018). 10.1038/nature25747 29469093 10.1038/nature25747

[45] Y.S. Ang, L. Cao, L.K. Ang, Physics of electron emission and injection in two-dimensional materials: theory and simulation. InfoMat **3**, 502–535 (2021). 10.1002/inf2.12168

[46] D. Akinwande, C. Huyghebaert, C.H. Wang, M.I. Serna, S. Goossens et al., Graphene and two-dimensional materials for silicon technology. Nature **573**, 507–518 (2019). 10.1038/s41586-019-1573-9 31554977 10.1038/s41586-019-1573-9

[47] L. Sun, Y. Zhang, G. Han, G. Hwang, J. Jiang et al., Self-selective van der Waals heterostructures for large scale memory array. Nat. Commun. **10**, 3161 (2019). 10.1038/s41467-019-11187-9 31320651 10.1038/s41467-019-11187-9 PMC6639341

[48] M. Wang, S. Cai, C. Pan, C. Wang, X. Lian et al., Robust memristors based on layered two-dimensional materials. Nat. Electron. **1**, 130–136 (2018). 10.1038/s41928-018-0021-4

[49] C.-Y. Wang, S.-J. Liang, S. Wang, P. Wang, Z.-A. Li et al., Gate-tunable van der Waals heterostructure for reconfigurable neural network vision sensor. Sci. Adv. **6**, eaba6173 (2020). 10.1126/sciadv.aba6173 32637614 10.1126/sciadv.aba6173 PMC7314516

[50] S. Seo, S.H. Jo, S. Kim, J. Shim, S. Oh et al., Artificial optic-neural synapse for colored and color-mixed pattern recognition. Nat. Commun. **9**, 5106 (2018). 10.1038/s41467-018-07572-5 30504804 10.1038/s41467-018-07572-5 PMC6269540

[51] K. Zhu, X. Liang, B. Yuan, M.A. Villena, C. Wen et al., Graphene-boron nitride-graphene cross-point memristors with three stable resistive states. ACS Appl. Mater. Interfaces **11**, 37999–38005 (2019). 10.1021/acsami.9b04412 31529969 10.1021/acsami.9b04412

[52] C. Choi, J. Leem, M. Kim, A. Taqieddin, C. Cho et al., Curved neuromorphic image sensor array using a MoS_2_-organic heterostructure inspired by the human visual recognition system. Nat. Commun. **11**, 5934 (2020). 10.1038/s41467-020-19806-6 33230113 10.1038/s41467-020-19806-6 PMC7683533

[53] L. Mennel, J. Symonowicz, S. Wachter, D.K. Polyushkin, A.J. Molina-Mendoza et al., Ultrafast machine vision with 2D material neural network image sensors. Nature **579**, 62–66 (2020). 10.1038/s41586-020-2038-x 32132692 10.1038/s41586-020-2038-x

[54] L. Chen, Z.G. Yu, D. Liang, S. Li, W.C. Tan et al., Ultrasensitive and robust two-dimensional indium selenide flexible electronics and sensors for human motion detection. Nano Energy **76**, 105020 (2020). 10.1016/j.nanoen.2020.105020

[55] S. Chen, M.R. Mahmoodi, Y. Shi, C. Mahata, B. Yuan et al., Wafer-scale integration of two-dimensional materials in high-density memristive crossbar arrays for artificial neural networks. Nat. Electron. **3**, 638–645 (2020). 10.1038/s41928-020-00473-w

[56] M. Sivan, Y. Li, H. Veluri, Y. Zhao, B. Tang et al., All WSe_2_ 1T1R resistive RAM cell for future monolithic 3D embedded memory integration. Nat. Commun. **10**, 5201 (2019). 10.1038/s41467-019-13176-4 31729375 10.1038/s41467-019-13176-4 PMC6858359

[57] C.-Y. Wang, C. Wang, F. Meng, P. Wang, S. Wang et al., 2D layered materials for memristive and neuromorphic applications. Adv. Electron. Mater. **6**, 1901107 (2020). 10.1002/aelm.201901107

[58] G. Cao, P. Meng, J. Chen, H. Liu, R. Bian et al., 2D material based synaptic devices for neuromorphic computing. Adv. Funct. Mater. **31**, 2005443 (2021). 10.1002/adfm.202005443

[59] Z. Zhang, D. Yang, H. Li, C. Li, Z. Wang et al., 2D materials and van der Waals heterojunctions for neuromorphic computing. Neuromorph. Comput. Eng. **2**, 032004 (2022). 10.1088/2634-4386/ac8a6a

[60] G. Lee, J.-H. Baek, F. Ren, S.J. Pearton, G.-H. Lee et al., Artificial neuron and synapse devices based on 2D materials. Small **17**, 2100640 (2021). 10.1002/smll.202100640 10.1002/smll.202100640 33817985

[61] K. Liao, P. Lei, M. Tu, S. Luo, T. Jiang et al., Memristor based on inorganic and organic two-dimensional materials: mechanisms, performance, and synaptic applications. ACS Appl. Mater. Interfaces **13**, 32606–32623 (2021). 10.1021/acsami.1c07665 34253011 10.1021/acsami.1c07665

[62] J. Bian, Z. Cao, P. Zhou, Neuromorphic computing: devices, hardware, and system application facilitated by two-dimensional materials. Appl. Phys. Rev. **8**, 041313 (2021). 10.1063/5.0067352

[63] F. Zhang, C. Li, Z. Li, L. Dong, J. Zhao, Recent progress in three-terminal artificial synapses based on 2D materials: from mechanisms to applications. Microsyst. Nanoeng. **9**, 16 (2023). 10.1038/s41378-023-00487-2 36817330 10.1038/s41378-023-00487-2 PMC9935897

[64] X. Liu, Z. Zeng, Memristor crossbar architectures for implementing deep neural networks. Complex Intell. Syst. **8**, 787–802 (2022). 10.1007/s40747-021-00282-4

[65] D. Ielmini, H.-S.P. Wong, In-memory computing with resistive switching devices. Nat. Electron. **1**, 333–343 (2018). 10.1038/s41928-018-0092-2

[66] S. Yu, H.Y. Chen, B. Gao, J. Kang, H.S. Wong, HfO_x_-based vertical resistive switching random access memory suitable for bit-cost-effective three-dimensional cross-point architecture. ACS Nano **7**, 2320–2325 (2013). 10.1021/nn305510u 23411406 10.1021/nn305510u

[67] C.-H. Yeh, D. Zhang, W. Cao, K. Banerjee, 0.5T0.5R - introducing an ultra-compact memory cell enabled by shared graphene edge-contact and h-BN insulator, in *2020 IEEE International Electron Devices Meeting (IEDM)*. San Francisco, CA, USA. IEEE, (2020)., 12.3.1–12.3.4

[68] H.-S. Lee, V.K. Sangwan, W.A.G. Rojas, H. Bergeron, H.Y. Jeong et al., Dual-gated MoS2 memtransistor crossbar array. Adv. Funct. Mater. **30**, 2003683 (2020). 10.1002/adfm.202003683

[69] J. Xie, S. Afshari, I. SanchezEsqueda, Hexagonal boron nitride (h-BN) memristor arrays for analog-based machine learning hardware npj 2D Mater. Appl. **6**, 50 (2022). 10.1038/s41699-022-00328-2

[70] M. Naqi, M.S. Kang, N. liu, T. Kim, S. Baek, et al., Multilevel artificial electronic synaptic device of direct grown robust MoS_2_ based memristor array for in-memory deep neural network npj 2D Mater. Appl. **6**, 53 (2022). 10.1038/s41699-022-00325-5

[71] S. Li, M.-E. Pam, Y. Li, L. Chen, Y.-C. Chien et al., Wafer-scale 2D hafnium diselenide based memristor crossbar array for energy-efficient neural network hardware. Adv. Mater. **34**, e2103376 (2022). 10.1002/adma.202103376 34510567 10.1002/adma.202103376

[72] Y. Shi, X. Liang, B. Yuan, V. Chen, H. Li et al., Electronic synapses made of layered two-dimensional materials. Nat. Electron. **1**, 458–465 (2018). 10.1038/s41928-018-0118-9

[73] Y. Li, L. Loh, S. Li, L. Chen, B. Li et al., Anomalous resistive switching in memristors based on two-dimensional palladium diselenide using heterophase grain boundaries. Nat. Electron. **4**, 348–356 (2021). 10.1038/s41928-021-00573-1

[74] M.A. Villena, F. Hui, X. Liang, Y. Shi, B. Yuan et al., Variability of metal/h-BN/metal memristors grown via chemical vapor deposition on different materials. Microelectron. Reliab. **102**, 113410 (2019). 10.1016/j.microrel.2019.113410

[75] J.B. Roldan, D. Maldonado, C. Aguilera-Pedregosa, F.J. Alonso, Y. Xiao et al., Modeling the variability of Au/Ti/h-BN/Au memristive devices. IEEE Trans. Electron Devices **70**, 1533–1539 (2023). 10.1109/TED.2022.3197677

[76] M.E. Pam, S. Li, T. Su, Y.C. Chien, Y. Li et al., Interface-modulated resistive switching in Mo-irradiated ReS_2_ for neuromorphic computing. Adv. Mater. **34**, e2202722 (2022). 10.1002/adma.202202722 35610176 10.1002/adma.202202722

[77] L. Wang, W. Liao, S.L. Wong, Z.G. Yu, S. Li et al., Artificial synapses based on multiterminal memtransistors for neuromorphic application. Adv. Funct. Mater. **29**, 1901106 (2019). 10.1002/adfm.201901106

[78] S. Li, B. Li, X. Feng, L. Chen, Y. Li et al., Electron-beam-irradiated rhenium disulfide memristors with low variability for neuromorphic computing npj 2D Mater. Appl. **5**, 1 (2021). 10.1038/s41699-020-00190-0

[79] J. Jadwiszczak, D. Keane, P. Maguire, C.P. Cullen, Y. Zhou et al., MoS_2_ memtransistors fabricated by localized helium ion beam irradiation. ACS Nano **13**, 14262–14273 (2019). 10.1021/acsnano.9b07421 31790198 10.1021/acsnano.9b07421

[80] D. Li, B. Wu, X. Zhu, J. Wang, B. Ryu et al., MoS_2_ memristors exhibiting variable switching characteristics toward biorealistic synaptic emulation. ACS Nano **12**, 9240–9252 (2018). 10.1021/acsnano.8b03977 30192507 10.1021/acsnano.8b03977

[81] L. Sun, Z. Wang, J. Jiang, Y. Kim, B. Joo et al., In-sensor reservoir computing for language learning via two-dimensional memristors. Sci. Adv. **7**, 1455 (2021). 10.1126/sciadv.abg1455 10.1126/sciadv.abg1455 PMC8121431 33990331

[82] G. Moon, S.Y. Min, C. Han, S.H. Lee, H. Ahn et al., Atomically thin synapse networks on van der Waals photo-memtransistors. Adv. Mater. **35**, e2203481 (2023). 10.1002/adma.202203481 35953281 10.1002/adma.202203481

[83] X. Zhu, D. Li, X. Liang, W.D. Lu, Ionic modulation and ionic coupling effects in MoS_2_ devices for neuromorphic computing. Nat. Mater. **18**, 141–148 (2019). 10.1038/s41563-018-0248-5 30559410 10.1038/s41563-018-0248-5

[84] N.T. Duong, Y.-C. Chien, H. Xiang, S. Li, H. Zheng et al., Dynamic ferroelectric transistor-based reservoir computing for spatiotemporal information processing. Adv. Intell. Syst. **5**, 2300009 (2023). 10.1002/aisy.202300009

[85] K. Liu, B. Dang, T. Zhang, Z. Yang, L. Bao et al., Multilayer reservoir computing based on ferroelectric α-In_2_ Se_3_ for hierarchical information processing. Adv. Mater. **34**, e2108826 (2022). 10.1002/adma.202108826 35064981 10.1002/adma.202108826

[86] X. Feng, S. Li, S.L. Wong, S. Tong, L. Chen et al., Self-selective multi-terminal memtransistor crossbar array for In-memory computing. ACS Nano **15**, 1764–1774 (2021). 10.1021/acsnano.0c09441 33443417 10.1021/acsnano.0c09441

[87] J.-J. Huang, Y.-M. Tseng, W.-C. Luo, C.-W. Hsu, T.-H. Hou, One selector-one resistor (1S1R) crossbar array for high-density flexible memory applications, in *2011 International Electron Devices Meeting*. Washington, DC, USA. IEEE, (2011)., 31.7.1–31.7.4

[88] K. Zhang, Y. Feng, F. Wang, Z. Yang, J. Wang, Two dimensional hexagonal boron nitride (2D-hBN): synthesis, properties and applications. J. Mater. Chem. C **5**, 11992–12022 (2017). 10.1039/C7TC04300G

[89] K. Zhu, S. Pazos, F. Aguirre, Y. Shen, Y. Yuan et al., Hybrid 2D-CMOS microchips for memristive applications. Nature **618**, 57–62 (2023). 10.1038/s41586-023-05973-1 36972685 10.1038/s41586-023-05973-1 PMC10232361

[90] B. Tang, H. Veluri, Y. Li, Z.G. Yu, M. Waqar et al., Wafer-scale solution-processed 2D material analog resistive memory array for memory-based computing. Nat. Commun. **13**, 3037 (2022). 10.1038/s41467-022-30519-w 35650181 10.1038/s41467-022-30519-w PMC9160094

[91] R. Yue, A.T. Barton, H. Zhu, A. Azcatl, L.F. Pena et al., HfSe_2_ thin films: 2D transition metal dichalcogenides grown by molecular beam epitaxy. ACS Nano **9**, 474–480 (2015). 10.1021/nn5056496 25496648 10.1021/nn5056496

[92] M.J. Mleczko, C. Zhang, H.R. Lee, H.H. Kuo, B. Magyari-Köpe et al., HfSe_2_ and ZrSe_2_: two-dimensional semiconductors with native high-κ oxides. Sci. Adv. **3**, e1700481 (2017). 10.1126/sciadv.1700481 28819644 10.1126/sciadv.1700481 PMC5553816

[93] V.G. Pleshchev, N.V. Selezneva, N.V. Baranov, Influence of copper intercalation on the resistive state of compounds in the Cu-HfSe_2_ system. Phys. Solid State **54**, 716–721 (2012). 10.1134/S1063783412040221

[94] V.G. Pleshchev, N.V. Melnikova, N.V. Baranov, Relaxation processes in an alternating-current electric field and energy loss mechanisms in hafnium diselenide cointercalated with copper and silver atoms. Phys. Solid State **58**, 1758–1763 (2016). 10.1134/S1063783416090274

[95] L. Liu, Y. Li, X. Huang, J. Chen, Z. Yang et al., Low-power memristive logic device enabled by controllable oxidation of 2D HfSe_2_ for In-memory computing. Adv. Sci. **8**, e2005038 (2021). 10.1002/advs.202005038 10.1002/advs.202005038 PMC8336485 34050639

[96] Y. Wang, F. Wu, X. Liu, J. Lin, J.-Y. Chen et al., High on/off ratio black phosphorus based memristor with ultra-thin phosphorus oxide layer. Appl. Phys. Lett. **115**, 193503 (2019). 10.1063/1.5115531

[97] H. Zhou, V. Sorkin, S. Chen, Z. Yu, K.-W. Ang et al., Design-dependent switching mechanisms of schottky-barrier-modulated memristors based on 2D semiconductor. Adv. Electron. Mater. **9**, 2201252 (2023). 10.1002/aelm.202201252

[98] Q. Fang, X. Zhao, C. Xia, F. Ma, Interfacial defect engineering on electronic states and electrical properties of MoS_2_/metal contacts. J. Alloys Compd. **864**, 158134 (2021). 10.1016/j.jallcom.2020.158134

[99] W.S. Yun, J.D. Lee, Schottky barrier tuning of the single-layer MoS_2_ on magnetic metal substrates through vacancy defects and hydrogenation. Phys. Chem. Chem. Phys. **18**, 31027–31032 (2016). 10.1039/C6CP05384J 27808310 10.1039/c6cp05384j

[100] J. Yuan, S.E. Liu, A. Shylendra, W.A. Gaviria Rojas, S. Guo et al., Reconfigurable MoS_2_ memtransistors for continuous learning in spiking neural networks. Nano Lett. **21**, 6432–6440 (2021). 10.1021/acs.nanolett.1c00982 34283622 10.1021/acs.nanolett.1c00982

[101] L. Tong, Z. Peng, R. Lin, Z. Li, Y. Wang et al., 2D materials-based homogeneous transistor-memory architecture for neuromorphic hardware. Science **373**, 1353–1358 (2021). 10.1126/science.abg3161 34413170 10.1126/science.abg3161

[102] A. Sebastian, R. Pendurthi, A. Kozhakhmetov, N. Trainor, J.A. Robinson et al., Two-dimensional materials-based probabilistic synapses and reconfigurable neurons for measuring inference uncertainty using Bayesian neural networks. Nat. Commun. **13**, 6139 (2022). 10.1038/s41467-022-33699-7 36253370 10.1038/s41467-022-33699-7 PMC9576759

[103] S. Hao, X. Ji, S. Zhong, K.Y. Pang, K.G. Lim et al., A monolayer leaky integrate-and-fire neuron for 2D memristive neuromorphic networks. Adv. Electron. Mater. **6**, 1901335 (2020). 10.1002/aelm.201901335

[104] K. Liu, T. Zhang, B. Dang, L. Bao, L. Xu et al., An optoelectronic synapse based on α-In_2_Se_3_ with controllable temporal dynamics for multimode and multiscale reservoir computing. Nat. Electron. **5**, 761–773 (2022). 10.1038/s41928-022-00847-2

[105] F. Miao, J. JoshuaYang, I. Valov, Y. Chai, Editorial: focus issue on 2D materials for neuromorphic computing. Neuromorph. Comput. Eng. **3**, 010201 (2023). 10.1088/2634-4386/acba3f

[106] R. Hasan, T.M. Taha, C. Yakopcic, On-chip training of memristor crossbar based multi-layer neural networks. Microelectron. J. **66**, 31–40 (2017). 10.1016/j.mejo.2017.05.005

[107] Y. Shen, W. Zheng, K. Zhu, Y. Xiao, C. Wen et al., Variability and yield in h-BN-based memristive circuits: the role of each type of defect. Adv. Mater. **33**, e2103656 (2021). 10.1002/adma.202103656 34480775 10.1002/adma.202103656

[108] The International Roadmap For Devices and Systems: 2022, https://irds.ieee.org/images/files/pdf/2022/2022IRDS_BC.pdf. Accessed 8 Nov 22

[109] B. Yuan, X. Liang, L. Zhong, Y. Shi, F. Palumbo et al., 150nm × 200nm cross-point hexagonal boron nitride-based memristors. Adv. Electron. Mater. **6**, 1900115 (2020). 10.1002/aelm.201900115

[110] Y. Zheng, H. Ravichandran, T.F. Schranghamer, N. Trainor, J.M. Redwing et al., Hardware implementation of Bayesian network based on two-dimensional memtransistors. Nat. Commun. **13**, 5578 (2022). 10.1038/s41467-022-33053-x 36151079 10.1038/s41467-022-33053-x PMC9508127

[111] A. Krishnaprasad, D. Dev, S.S. Han, Y. Shen, H.S. Chung et al., MoS_2_ synapses with ultra-low variability and their implementation in Boolean logic. ACS Nano **16**, 2866–2876 (2022). 10.1021/acsnano.1c09904 35143159 10.1021/acsnano.1c09904

[112] J.B. Roldan, D. Maldonado, C. Aguilera-Pedregosa, E. Moreno, F. Aguirre et al. Spiking neural networks based on two-dimensional materials npj 2D Mater. Appl. **6**, 63 (2022). 10.1038/s41699-022-00341-5

[113] K. Wang, L. Li, R. Zhao, J. Zhao, Z. Zhou et al., A pure 2H-MoS_2_ nanosheet-based memristor with low power consumption and linear multilevel storage for artificial synapse emulator. Adv. Electron. Mater. **6**, 1901342 (2020). 10.1002/aelm.201901342

[114] M. Lanza, G. Molas, I. Naveh, The gap between academia and industry in resistive switching research. Nat. Electron. **6**, 260–263 (2023). 10.1038/s41928-023-00954-8

[115] Y.-C. Chien, H. Xiang, J. Wang, Y. Shi, X. Fong et al., Attack resilient true random number generators using ferroelectric-enhanced stochasticity in 2D transistor. Small **19**, e2302842 (2023). 10.1002/smll.202302842 37194958 10.1002/smll.202302842

[116] X. Chen, T. Wang, J. Shi, W. Lv, Y. Han et al., A novel artificial neuron-like gas sensor constructed from CuS quantum dots/Bi_2_S_3_ nanosheets. Nano-Micro Lett. **14**, 8 (2021). 10.1007/s40820-021-00740-1 10.1007/s40820-021-00740-1 PMC8639894 34859321

[117] S.A. Van, Building blocks for electronic spiking neural networks. Neural Netw. **14**, 617–628 (2001). 10.1016/s0893-6080(01)00067-3 11665758 10.1016/s0893-6080(01)00067-3

[118] D. Dev, A. Krishnaprasad, M.S. Shawkat, Z. He, S. Das et al., 2D MoS_2_-based threshold switching memristor for artificial neuron. IEEE Electron Device Lett. **41**, 936–939 (2020). 10.1109/LED.2020.2988247

[119] Z. Zhang, S. Gao, Z. Li, Y. Xu, R. Yang et al., Artificial LIF neuron with bursting behavior based on threshold switching device. IEEE Trans. Electron Devices **70**, 1374–1379 (2023). 10.1109/TED.2023.3236906

[120] H. Kalita, A. Krishnaprasad, N. Choudhary, S. Das, D. Dev et al., Artificial neuron using vertical MoS_2_/graphene threshold switching memristors. Sci. Rep. **9**, 53 (2019). 10.1038/s41598-018-35828-z 30631087 10.1038/s41598-018-35828-z PMC6328611

[121] A. Dodda, N. Trainor, J.M. Redwing, S. Das, All-in-one, bio-inspired, and low-power crypto engines for near-sensor security based on two-dimensional memtransistors. Nat. Commun. **13**, 3587 (2022). 10.1038/s41467-022-31148-z 35739100 10.1038/s41467-022-31148-z PMC9226122

[122] S. Fu, J.-H. Park, H. Gao, T. Zhang, X. Ji et al., Two-terminal MoS_2_ memristor and the homogeneous integration with a MoS_2_ transistor for neural networks. Nano Lett. **23**, 5869–5876 (2023). 10.1021/acs.nanolett.2c05007 37338212 10.1021/acs.nanolett.2c05007

[123] P. Yao, H. Wu, B. Gao, J. Tang, Q. Zhang et al., Fully hardware-implemented memristor convolutional neural network. Nature **577**, 641–646 (2020). 10.1038/s41586-020-1942-4 31996818 10.1038/s41586-020-1942-4

[124] X. Wang, P. Xie, B. Chen, X. Zhang, Chip-based high-dimensional optical neural network. Nano-Micro Lett. **14**, 221 (2022). 10.1007/s40820-022-00957-8 36374430 10.1007/s40820-022-00957-8 PMC9663775

[125] Z. Wang, S. Joshi, S. Savel’ev, W. Song, R. Midya, et al., Fully memristive neural networks for pattern classification with unsupervised learning. Nat. Electron. **1**, 137–145 (2018). 10.1038/s41928-018-0023-2

[126] Y. Wang, W. Gao, S. Yang, Q. Chen, C. Ye et al., Humanoid intelligent display platform for audiovisual interaction and sound identification. Nano-Micro Lett. **15**, 221 (2023). 10.1007/s40820-023-01199-y 10.1007/s40820-023-01199-y PMC10562358 37812331

[127] Y. Qiao, J. Luo, T. Cui, H. Liu, H. Tang et al., Soft electronics for health monitoring assisted by machine learning. Nano-Micro Lett. **15**, 66 (2023). 10.1007/s40820-023-01029-1 10.1007/s40820-023-01029-1 PMC10014415 36918452

[128] R. Wu, S. Seo, L. Ma, J. Bae, T. Kim, Full-fiber auxetic-interlaced yarn sensor for sign-language translation glove assisted by artificial neural network. Nano-Micro Lett. **14**, 139 (2022). 10.1007/s40820-022-00887-5 10.1007/s40820-022-00887-5 PMC9249965 35776226

[129] S.W. Cho, C. Jo, Y.H. Kim, S.K. Park, Progress of materials and devices for neuromorphic vision sensors. Nano-Micro Lett. **14**, 203 (2022). 10.1007/s40820-022-00945-y 10.1007/s40820-022-00945-y PMC9569410 36242681

[130] Z. Shi, L. Meng, X. Shi, H. Li, J. Zhang et al., Morphological engineering of sensing materials for flexible pressure sensors and artificial intelligence applications. Nano-Micro Lett. **14**, 141 (2022). 10.1007/s40820-022-00874-w 10.1007/s40820-022-00874-w PMC9256895 35789444

[131] J. Zeng, J. Zhao, T. Bu, G. Liu, Y. Qi et al., A flexible tribotronic artificial synapse with bioinspired neurosensory behavior. Nano-Micro Lett. **15**, 18 (2022). 10.1007/s40820-022-00989-0 10.1007/s40820-022-00989-0 PMC9800681 36580114

[132] K.C. Kwon, J.H. Baek, K. Hong, S.Y. Kim, H.W. Jang, Memristive devices based on two-dimensional transition metal chalcogenides for neuromorphic computing. Nano-Micro Lett. **14**, 58 (2022). 10.1007/s40820-021-00784-3 10.1007/s40820-021-00784-3 PMC8818077 35122527

[133] K. Simonyan, A. Zisserman, Very deep convolutional networks for large-scale image recognition, arXiv:1409.1556.

[134] J. Redmon, S. Divvala, R. Girshick, A. Farhadi, You only look once: unified, real-time object detection, in *2016 IEEE Conference on Computer Vision and Pattern Recognition (CVPR)*. Las Vegas, NV, USA. IEEE, (2016), 779–788. 10.1109/CVPR.2016.91

[135] H. Chen, T. Wan, Y. Zhou, J. Yan, C. Chen et al., Highly nonlinear memory selectors with ultrathin MoS_2_/WSe_2_/MoS_2_ heterojunction. Adv. Funct. Mater. (2023). 10.1002/adfm.202304242

[136] R. Midya, Z. Wang, J. Zhang, S.E. Savel’ev, C. Li et al., Anatomy of Ag/*Hafnia*-based selectors with 10^10^ nonlinearity. Adv. Mater. **29**, 1604457 (2017). 10.1002/adma.201604457 10.1002/adma.201604457 28134458

